# Pelle Modulates dFoxO-Mediated Cell Death in *Drosophila*

**DOI:** 10.1371/journal.pgen.1005589

**Published:** 2015-10-16

**Authors:** Chenxi Wu, Yujun Chen, Feng Wang, Changyan Chen, Shiping Zhang, Chaojie Li, Wenzhe Li, Shian Wu, Lei Xue

**Affiliations:** 1 Department of Interventional Radiology, Shanghai 10th People's Hospital, Shanghai Key Laboratory of Signaling and Disease Research, School of Life Science and Technology, Tongji University, Shanghai, China; 2 State Key Laboratory of Medicinal Chemical Biology and College of Life Sciences, Nankai University, Tianjin, China; Harvard Medical School, UNITED STATES

## Abstract

Interleukin-1 receptor-associated kinases (IRAKs) are crucial mediators of the IL-1R/TLR signaling pathways that regulate the immune and inflammation response in mammals. Recent studies also suggest a critical role of IRAKs in tumor development, though the underlying mechanism remains elusive. Pelle is the sole *Drosophila* IRAK homolog implicated in the conserved Toll pathway that regulates Dorsal/Ventral patterning, innate immune response, muscle development and axon guidance. Here we report a novel function of *pll* in modulating apoptotic cell death, which is independent of the Toll pathway. We found that loss of *pll* results in reduced size in wing tissue, which is caused by a reduction in cell number but not cell size. Depletion of *pll* up-regulates the transcription of pro-apoptotic genes, and triggers caspase activation and cell death. The transcription factor dFoxO is required for loss-of-*pll* induced cell death. Furthermore, loss of *pll* activates dFoxO, promotes its translocation from cytoplasm to nucleus, and up-regulates the transcription of its target gene *Thor/4E-BP*. Finally, Pll physically interacts with dFoxO and phosphorylates dFoxO directly. This study not only identifies a previously unknown physiological function of *pll* in cell death, but also shed light on the mechanism of IRAKs in cell survival/death during tumorigenesis.

## Author Summary

In the present study we report a Toll pathway independent function of Pll in modulating apoptotic cell death. Our major findings include: 1) loss of *pll* generates Toll pathway-independent wing phenotypes, which are caused by a reduction in cell number but not cell size; 2) depletion of *pll* up-regulates the transcription of pro-apoptotic genes *hid*, *reaper* and *grim*, and triggers caspase-mediated cell death, whereas cell proliferation remains unaffected; 3) *pll* modulates caspase activation and cell death through the transcription factor dFoxO; 4) loss of *pll* promotes dFoxO translocation from cytoplasm to nucleus, and activates the transcription of dFoxO target gene *Thor/4E-BP*; 5) Pll physically interacts with dFoxO and phosphorylates dFoxO directly. Thus, this work characterizes a previously unknown physiological function of *pll* that is independent of the Toll pathway.

## Introduction

Interleukin-1 receptor-associated kinases (IRAKs) are evolutionarily conserved Serine/Threonine (Ser/Thr) kinases that modulates Toll-like receptors (TLRs) and interleukin-1 receptors (IL-1Rs) signaling in the immune and inflammation response [1–3]. IRAKs transduce TLR/IL-1R signals to regulate subcellular distribution and activity of NF**-**κB transcription factor family via highly conserved downstream signaling molecules, including IκB kinase (IκK) complex and IκB [4,5]. While there are four IRAK family members (IRAK-1, IRAK-2, IRAK-4 and IRAK-M) in mammals, Pelle (Pll) is the only IRAK homolog in *Drosophila* [6]. Although Tube was initially characterized as a *Drosophila* IRAK homolog, it lacks the catalytic domain and thus functions as an adaptor protein in the Toll pathway [7]. As such, *Drosophila melanogaster* offers unique opportunities to investigate the physiological functions of IRAK with reduced genetic redundancy.

The Toll pathway in *Drosophila* has been implicated as a crucial regulator in embryonic Dorsal/Ventral (D/V) patterning [8,9], innate immune response against Gram-positive bacteria and fungi [10], proper muscle development and axon guidance [11,12]. This pathway is equivalent to the mammalian IL-1R/TLR pathway, with Toll/Cactus/Dorsal representing the respective homologs of IL-1R/IκB/NF**-**κB [13]. However, the *Drosophila* IκK is not involved in the Toll pathway [14,15], and Pll has been shown to be the only kinase implicated in Cactus phosphorylation [6,16]. Besides its role in the conserved pathway, it remains unknown whether Pll performs any Toll pathway independent functions *in vivo*.

Programmed cell death (PCD) or apoptosis is an important physiological regulator for tissue homeostasis and correct development. Cancer cells generally obtain the ability to evade cell death signaling pathways, which act as a protective tumor suppressive mechanism to remove damaged cells from the tissue. The central roles of caspases (cysteine aspartate-specific proteinases) have been widely studied in the context of apoptosis, and the core machinery of caspases signaling pathway, including the initiator caspases and the effector caspases, has been conserved throughout evolution [17,18]. In *Drosophila*, apoptosis is activated by the intrinsic and extrinsic cell death signals through transcriptional up-regulation of three pro-apoptotic genes *reaper* (*rpr*), *head involution defective* (*hid*) and *grim* [19]. Rpr, Hid and Grim (RHG) proteins subsequently bind to and antagonize the *Drosophila* inhibitor of apoptosis protein 1 (DIAP1), which in turn dampens caspases [20,21]. DIAP1 functions as an E3-ubiquitin ligase to block cell death by targeting the initiator caspase Dronc (*Drosophila* caspase-9) for degradation, and thus suppresses the activation of the downstream effector caspase Drice (*Drosophila* caspase-3) [22,23]. Hence, apoptosis is the consequence of a train of complex interactions between RHG proteins, DIAP1 and caspases in *Drosophila*.

Forkhead Box O (FoxO) transcription factors were originally identified in C. *elegans* as DAF-16 for its role in aging, and have been highly conserved in *Drosophila* (dFoxO) and mammals (FoxO1, FoxO3a, FoxO4 and FoxO6) [24–28]. FoxO proteins are known to modulate various physiological processes including cell proliferation and differentiation, DNA repair, apoptosis, metabolism, oxidative stress and lifespan [25,26,28–33]. In general, the multiple functions of FoxO transcription factors are regulated by various post-translational modifications (PTMs), including methylation, acetylation, ubiquitination, glycosylation and phosphorylation [34]. For example, FoxOs are targeted for phosphorylation by a number of kinases in response to different cellular stresses, which in turn regulate the nuclear-cytoplasmic shuttling of FoxOs and their transcriptional activity [34]. In *Drosophila*, dFoxO was reported as a key modulator in the UV-induced caspase-mediated apoptotic response by directly regulating the expression of pro-apoptotic gene *hid* [30].

In this study, we examined the physiological function of *pelle* (*pll*) in *Drosophila* development, and found that *pll* negatively regulates FoxO-mediated apoptotic cell death *in vivo*. First, loss of *pll* in the developing wing generates Toll/NF**-**κB pathway independent phenotypes, which are caused by a reduction in cell number but not cell size. Secondly, depletion of *pll* triggers caspase-mediated cell death, whereas cell proliferation remains unaffected. Thirdly, *pll* modulates caspase activation and cell death through dFoxO. Fourthly, loss of *pll* results in activation of dFoxO, as manifested by its translocation from cytoplasm to nucleus and the expression of its target gene *Thor/4E-BP*. Finally, Pll physically interacts with and phosphorylates on dFoxO, which likely contributes to the cytoplasmic retention of dFoxO. Therefore, we have identified a previously uncharacterized physiological function of *pll* in cell death, which is independent of the canonical Toll/NF**-**κB pathway.

## Results

### Loss of *pll* produces Toll pathway-independent wing phenotype

**Fig 1:**
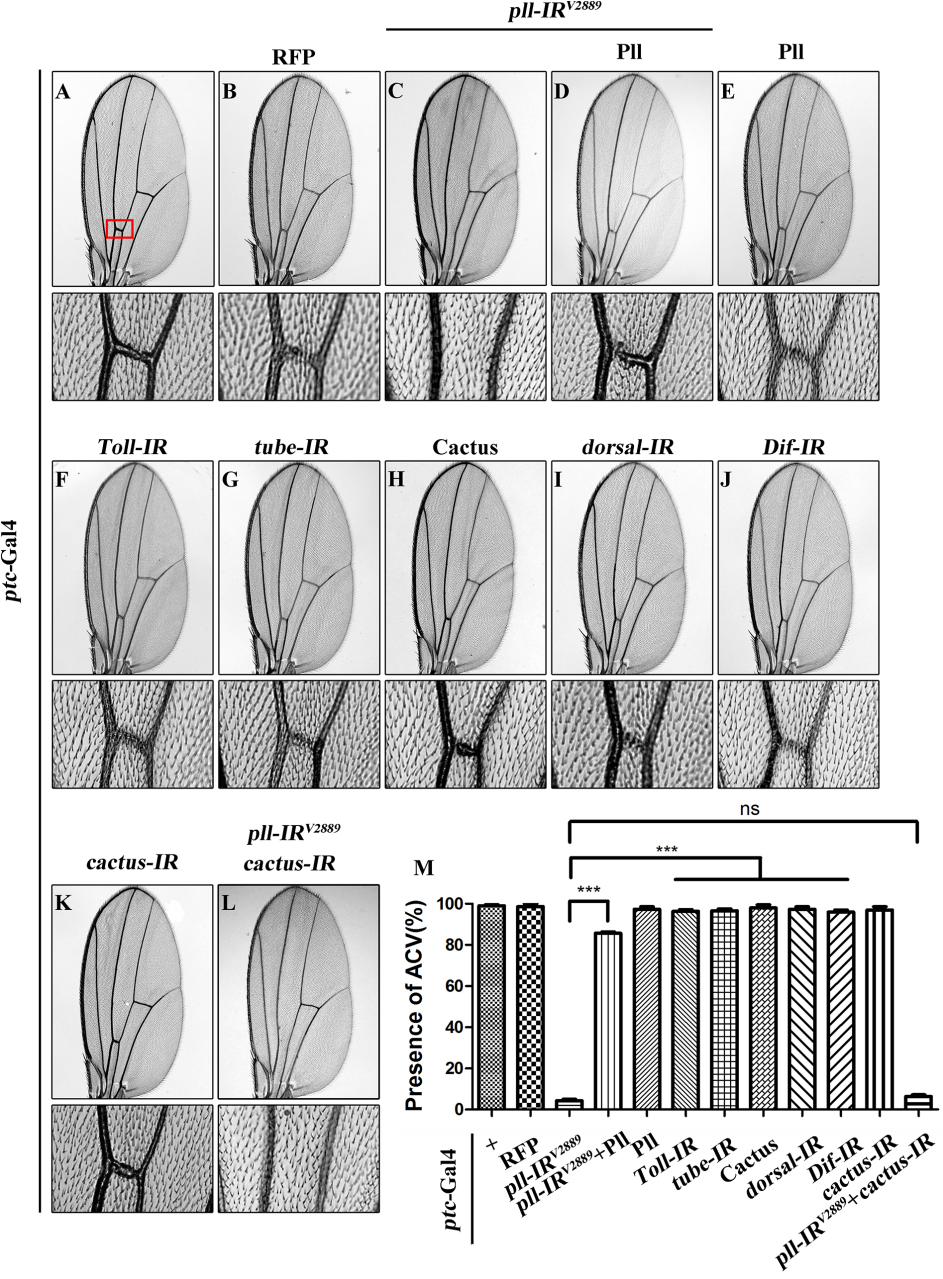
Loss of *pll* produces Toll/NF-κB pathway independent wing phenotype. (**A**-**L**) Light micrographs showing *Drosophila* adult wings, anterior is to the left and distal up. Compared with *ptc*-Gal4 control (**A**), expression of *pll* RNAi (*pll-IR* ^*V2889*^) induced a loss-of-ACV phenotype (**C**) that was rescued by expression of Pll (**D**), while expression of Pll alone showed no obvious defects (**E**). Expression of RFP was included as a negative control (**B**). Down-regulation of Toll/NF**-**κB pathway by expressing RNAi of *Toll* (**F**), *tube* (**G**), *dorsal* (**I**) or *Dif* (**J**), or the negative regulator Cactus (**H**), had no effect on wing development. *ptc>pll-IR* ^*V2889*^ was not rescued by co-expressing a *cactus* RNAi (**K** and **L**). The lower panels show high magnification view of the ACV area, boxed in panel A, in upper panels. (**M**) Quantification of the ACV phenotype as shown in figures **A**-**L**. One-way ANOVA with Bonferroni multiple comparison test was used to compute *P*-values, significance is indicated with asterisks (*** *P*<0.001). ns stands for not significant. Detailed genotypes: (A) *ptc*-Gal4/+ (B) *ptc*-Gal4/+; *UAS*-RFP/+ (C) *ptc*-Gal4/*UAS-pll-IR* ^*V2889*^ (D) *ptc*-Gal4/*UAS-pll-IR* ^*V2889*^; *UAS*-Pll; (E) *ptc*-Gal4/+; *UAS*-Pll/+ (F) *ptc*-Gal4/+; *UAS-Toll-IR*/+ (G) *ptc*-Gal4/+; *UAS-tube-IR*/+ (H) *ptc*-Gal4/*UAS*-Cactus (I) *ptc*-Gal4/+; *UAS-dorsal-IR*/+ (J) *ptc*-Gal4/+; *UAS-Dif-IR*/+ (K) *ptc*-Gal4/+; *UAS-cactus-IR*/+ (L) *ptc*-Gal4/*UAS-pll-IR* ^*V2889*^; *UAS-cactus-IR*/+.

As a crucial component of the Toll pathway, Pll is known to regulate the dorsal-ventral polarity in early embryos [35] and the immune response in fat body [10]. Despite its ubiquitous lower level expression throughout development [6], the post-embryonic functions of Pll remain poorly understood. To explore the physiological function of *pll* in late development, we employed the UAS/Gal4 binary system to knockdown *pll* in a specific temporal and spatial manner. We found that expression of a *pll* RNA interference (RNAi) driven by *ptc*-Gal4 (*ptc>pll-IR*) along the anterior/posterior (A/P) compartment boundary resulted in a consistent loss of anterior cross-vein (ACV) phenotype (Fig 1C and 1M), compared with *ptc*-Gal4 control (Fig 1A) or RFP expression (Fig 1B). To exclude the possibility that the phenotype is a result of RNAi’s off-target effect, we examined two additional *pll* RNAi that target distinct regions of the *pll* transcript, and observed the same loss-of-ACV phenotype (S1A, S1F and S1H Fig). In addition, this phenotype could be rescued by expression of Pll (Fig 1D and 1M; S1G and S1H Fig), which by itself has no effect on ACV development (Fig 1E and 1M). The knock-down efficiencies of three *pll* RNAi lines were verified by their abilities to suppress the *GMR*>Pll rough eye phenotype (S2A–S2E Fig) and by real-time quantitative reverse transcription polymerase chain reaction (qRT-PCR) assay (S2L Fig). To further verify the physiological function of *pll* in ACV development, we examined two *pll* mutant alleles: *pll* ^*2*^ and *pll* ^*7*^. While the heterozygous mutants show no obvious ACV defect, they could significantly increase the percentage of loss-of-ACV phenotype in the *ptc>pll-IR* ^*W*^ background (S1A–S1E and S1H Fig). Together these data suggest that *pll* is physiologically required for ACV development in *Drosophila* wing.

Pll has been implicated in the Toll/NF**-**κB pathway that directly phosphorylates the *Drosophila* IκB factor Cactus [36,37]. To examine whether the Toll/NF**-**κB pathway is required for the development of ACV, we down-regulated the pathway by knocking-down the positive regulators such as *Toll*, *tube*, *dorsal* and *Dif*, or ectopically expressing the negative regulator Cactus. At least two independent RNAi lines were tested for each gene, including a previously reported RNAi for *Toll* [38] and *tube* [39]. The knock-down efficacies of the RNAi lines were verified by their ability to suppress the rough eye phenotype of *GMR*>Toll^10B^ (S2F–S2K Fig), which expresses the activated form of Toll in the developing eye. Intriguingly, down-regulation of the Toll/NF**-**κB pathway failed to recapitulate the loss-of-ACV phenotype produced by *ptc>pll-IR* (Fig 1F–1J and 1M), and this phenotype was not rescued by RNAi inactivation of *cactus* (Fig 1K–1M), the *Drosophila* I**-**κB homolog, implying that the physiological function of *pll* in wing development is independent of the canonical Toll/NF-κB pathway.

### *pelle* regulates cell number, but not cell size in wing development

**Fig 2:**
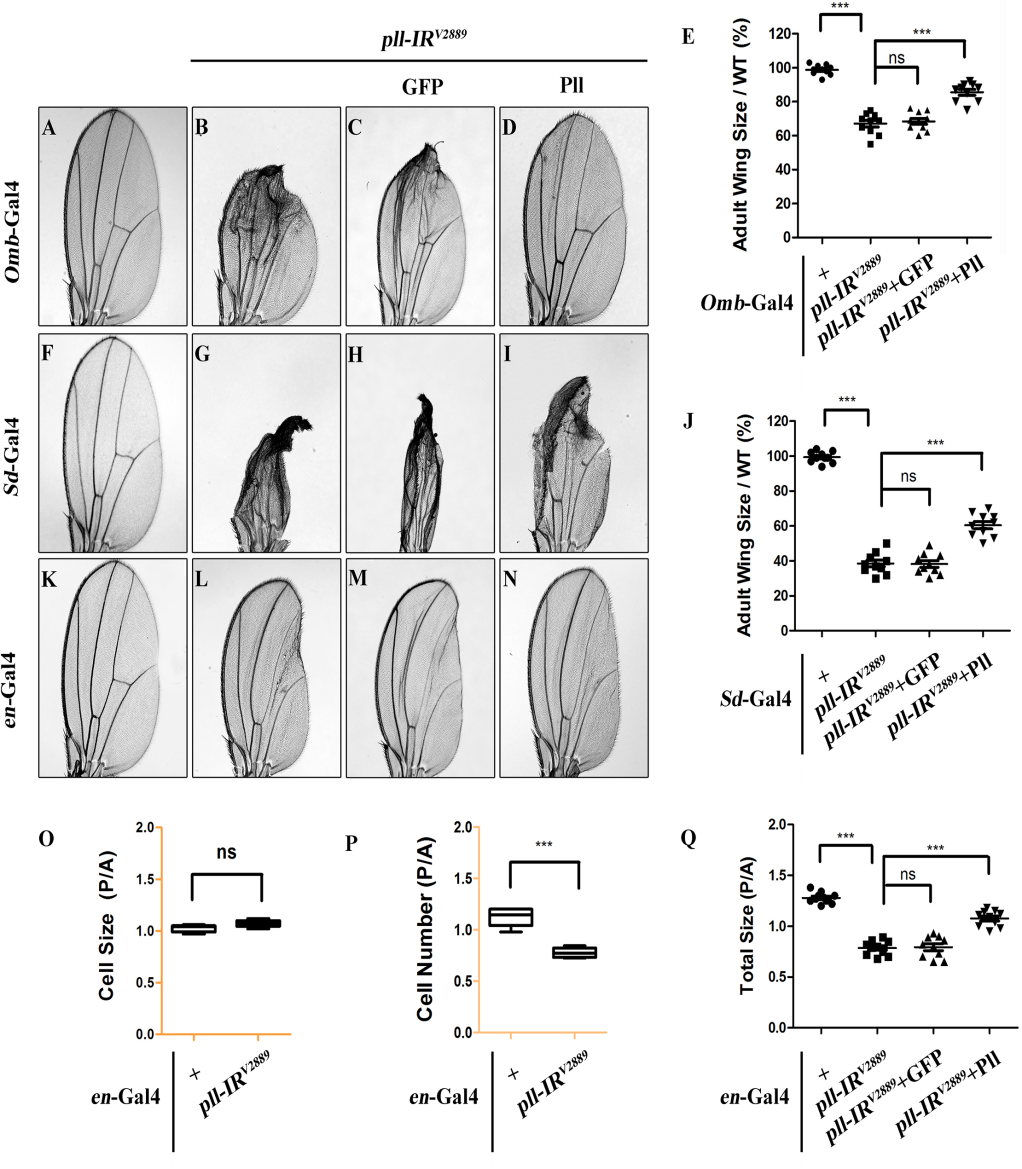
*pll* regulates cell number, but not cell size in adult wing. (**A**-**D**, **F**-**I** and **K**-**N**) Light micrographs of *Drosophila* adult wings are shown, anterior is to the left and distal up. Compared with the Gal4 controls (**A**, **F** and **K**), expression of *pll* RNAi (*pll-IR* ^*V2889*^) driven by *Omb*-Gal4 (**B**), *Sd*-Gal4 (**G**) or *en*-Gal4 (**L**) resulted in reduced wing tissue in the corresponding areas, which were rescued by expression of Pll (**D**, **I** and **N**), but not that of GFP (**C**, **H** and **M**). (**E** and **J**) Quantifications of adult wing size/wild type (WT) ratio are shown for figures **A**-**D** and **F**-**I** respectively (n = 10). One-way ANOVA with Bonferroni multiple comparison test was used to compute *P*-values, significance is indicated with asterisks (*** *P*<0.001). (**O** and **P**) Quantifications of cell size (**O**) and cell number (**P**) in wings shown in **K** and **L**. The P/A ratio of cell size showed no difference while that of cell number decreased significantly when *pll* was knocked down in the P compartment by *en*-Gal4. Unpaired t test was used to calculate statistical significance, indicated with asterisks (*** *P*<0.001, n = 10 in each group). (**Q**) Statistic analysis of total size P/A ratio are shown for figures **K-N**. One-way ANOVA with Bonferroni multiple comparison test was used to compute *P*-values, significance is indicated with asterisks (*** *P*<0.001). ns stands for not significant. Detailed genotypes: (A) *Omb-*Gal4/*+* (B) *Omb-*Gal4/*+*; *UAS-pll-IR* ^*V2889*^/+ (C) *Omb-*Gal4/*+*; *UAS-pll-IR* ^*V2889*^/+; *UAS-*GFP/+ (D) *Omb-*Gal4/*+*; *UAS-pll-IR* ^*V2889*^/+; *UAS-*Pll/+ (F) *Sd-*Gal4/*+* (G) *Sd-*Gal4/*+*; *UAS-pll-IR* ^*V2889*^/+ (H) *Sd-*Gal4/*+*; *UAS-pll-IR* ^*V2889*^/+; *UAS-*GFP/+ (I) *Sd-*Gal4/*+*; *UAS-pll-IR* ^*V2889*^/+; *UAS-*Pll/+ (K) *en*-Gal4/+ (L) *en*-Gal4/*UAS-pll-IR* ^*V2889*^ (M) *en*-Gal4/*UAS-pll-IR* ^*V2889*^; *UAS-*GFP/+ (N) *en*-Gal4/*UAS-pll-IR* ^*V2889*^; *UAS-*Pll/+.

To further characterize the physiological function of *pll* in wing development, we knocked down *pll* in distinct regions of the wing disc by using additional wing specific Gal4 drivers: *Optomotor-blind* (*Omb*)-Gal4, *Scalloped* (*Sd)*-Gal4 and *engrailed* (*en*)-Gal4. We noted that loss of *pll* in the distal part (*Omb>pll-IR*), wing pouch (*Sd*>*pll-IR*) or posterior compartment (*en*>*pll-IR*) of the wing disc caused severe reduction in corresponding areas of the adult wing, which were rescued by co-expression of Pll, but not that of GFP (Fig 2A–2N and 2Q; S3 Fig), confirming that *pll* is required for proper wing development.

**Fig 3:**
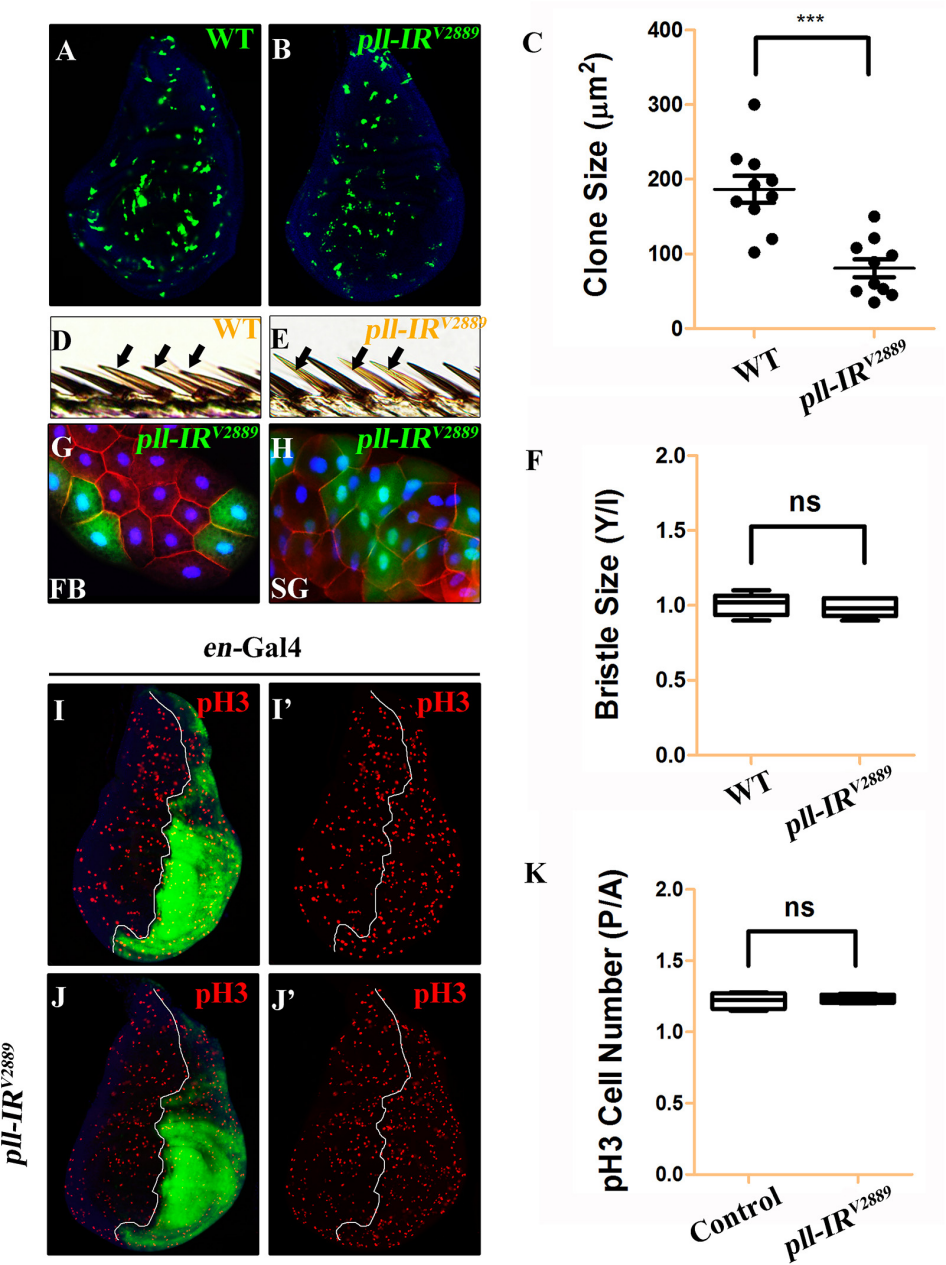
*pll* regulates cell number but not cell size in development. (**A** and **B**) Fluorescence micrographs of third instar larval wing discs with clones (marked by GFP) expressing no (**A**) or *pll* RNAi (**B**). Clones were induced by heat shock at 37°C for 1 hour and recovered at 25°C for 42 hours. Nuclei were labeled with DAPI (blue). (**C**) Quantification of clone size shown in **A** and **B**. Unpaired t test was used to calculate statistical significance, indicated with asterisks (*** *P*<0.001, n = 10 in each group). (**D** and **E**) Clones (marked by y^-^, yellow) expressing no (**D**) or *pll* RNAi (**E**) in adult wing margin bristles, 7 days after induction. (**F**) Quantification of bristle size as shown in **D** and **E**. The ratio of bristle size within clones (Y)/bristle size of internal control (I) remained unchanged when *pll* was knocked down in the clone. (**G** and **H**) Fluorescence micrographs of clones (marked by GFP) expressing *pll* RNAi in third instar larval fat body (FB, **G**) and salivary gland (SG, **H**) 42 hours after induction. Nuclei were labelled with DAPI (blue), cell membranes were stained by anti-Dlg antibody (red). (**I** and **J**) Fluorescence micrographs of wing discs are shown. The P compartment was labelled by GFP, while cell proliferation was detected by anti-pH3 staining (red) (**I’** and **J’**), nuclei were labelled with DAPI (blue). (**K**) Quantification of pH3 positive cells shown in **I** and **J**. The P/A ratio of pH3 positive cells remained unchanged when *pll* was knocked down in the P compartment. ns stands for not significant. For all wing discs, anterior is to the left and dorsal up. Detailed genotypes: (A and D) *y w hs*-Flp/+; *act*>y+>Gal4 *UAS*-GFP/+ (B, E, G, H) *y w hs*-Flp/+; *act*>y+>Gal4 *UAS*-GFP/*UAS-pll-IR* ^*V2889*^ (I) *en*-Gal4 *UAS-*GFP/+ (J) *en*-Gal4 *UAS-*GFP/*UAS-pll-IR* ^*V2889*^.

To investigate the underlying mechanism by which loss of *pll* results in reduced wing tissue, we compared the cell number and cell size in the posterior (P) compartment with that in the anterior (A) compartment of the adult wing. We found that when *pll* was specifically knock-down in the P compartment by *en*-Gal4 (Fig 2L), the P/A ratio of cell size remained unaffected (Fig 2O), whereas the P/A ratio of cell number and total size decreased significantly (Fig 2P and 2Q), suggesting that *pll* regulates cell number, but not cell size, in wing development. To further verify this conclusion, we generated *pll* loss-of-function clones in larvae and found that the size of clones, labeled by GFP expression, was significantly smaller than that of wild-type (WT) controls in wing discs (Fig 3A–3C), confirming the role of *pll* in regulating tissue growth. However, the sizes of cells inside the clones (marked by GFP expression) were similar to that of wild-type controls in fat body (FB) and salivary gland (SG) (Fig 3G and 3H). Furthermore, wing margin bristles in *pll* knock-down clones, marked by loss of the *yellow* (*y*) gene, are not statistically different in size from their wild-type neighbors (Fig 3D–3F). Together, these data indicate that *pll* modulates tissue growth via regulating cell number but not cell size.

### Loss of *pll* triggers caspase-mediated cell death

Loss of *pll* results in reduced cell number, which could be a consequence of decreased cell proliferation or increased cell death. To distinguish between the two possibilities, we first checked the cell proliferation rate by phospho-Histone H3 (pH3) staining in wing discs. We found that knock-down *pll* in the P compartment of wing discs (*en*>*pll-IR*) did not affect cell proliferation, as the P/A ratio of pH3 positive cells in such discs was not statistically different from that in control discs (Fig 3I–3K).

**Fig 4:**
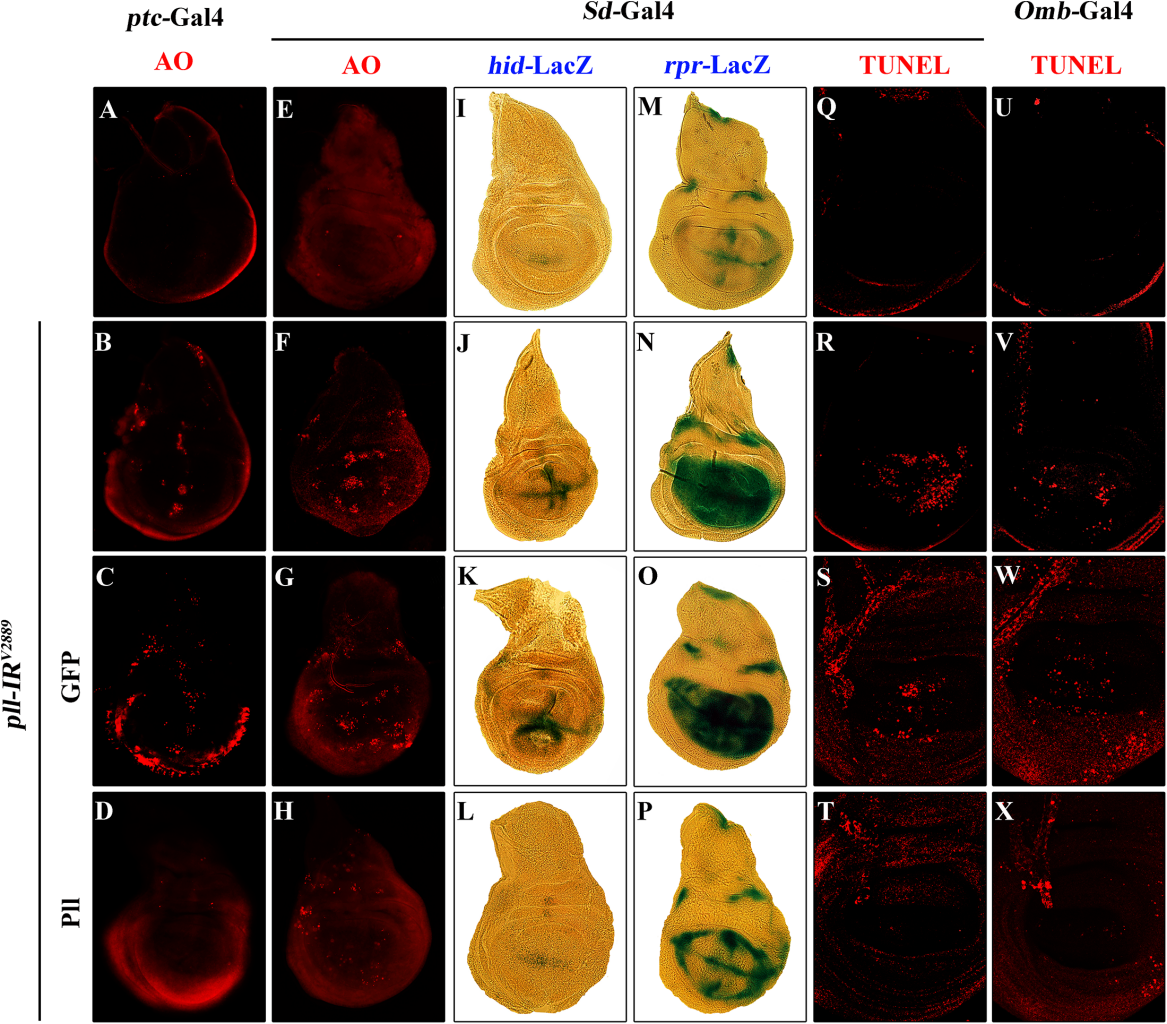
Loss of *pll* elicits apoptotic cell death. (**A-H**) AO staining of third instar larval wing discs. Compared with controls (**A** and **E**), knock down *pll* along the A/P boundary by *ptc*-gal4 (**B**) or in the wing pouch by *Sd*-Gal4 (**F**) triggered cell death in the corresponding areas, which was suppressed by expressing Pll (**D** and **H**), but not GFP (**C** and **G**). (**I-P**) X-Gal staining of a *hid*-LacZ and an *rpr*-LacZ reporters in wing discs. Compared with controls (**I** and **M**), knock down *pll* in the wing pouch induced *hid* (**J**) and *rpr* (**N**) transcription, which was blocked by expressing Pll (**L** and **P**), but not GFP (**K** and **O**). (**Q-X**) TUNEL staining of third instar larval wing discs. Compared with Gal4 controls (**Q** and **U**), knock down *pll* by *Sd*-Gal4 (**R**) or *Omb*-Gal4 (**V**) induced cell death in the corresponding areas, which was impeded by expressing Pll (**T** and **X**), but not GFP (**S** and **W**). In all figures, anterior is to the left and dorsal up. Detailed genotypes: (A) *ptc-*Gal4/*+* (B) *ptc-*Gal4/*UAS-pll-IR* ^*V2889*^ (C) *ptc-*Gal4/*UAS-pll-IR* ^*V2889*^; *UAS*-GFP/+ (D) *ptc-*Gal4/*UAS-pll-IR* ^*V2889*^; *UAS*-Pll/+ (E and Q) *Sd-*Gal4/*+* (F and R) *Sd-*Gal4/*+*; *UAS-pll-IR* ^*V2889*^/+ (G and S) *Sd-*Gal4/*+*; *UAS-pll-IR* ^*V2889*^/+; *UAS*-GFP/+ (H and T) *Sd-*Gal4/*+*; *UAS-pll-IR* ^*V2889*^/+; *UAS*-Pll/+ (I) *Sd-*Gal4/*+*; *hid*-LacZ/+ (J) *Sd-*Gal4/*+*; *UAS-pll-IR* ^*V2889*^/+; *hid*-LacZ/+ (K) *Sd-*Gal4/*+*; *UAS-pll-IR* ^*V2889*^/+; *hid*-LacZ/*UAS*-GFP (L) *Sd-*Gal4/*+*; *UAS-pll-IR* ^*V2889*^/+; *hid*-LacZ/*UAS*-Pll (M) *Sd-*Gal4/*+*; *rpr*-LacZ/+ (N) *Sd-*Gal4/*+*; *UAS-pll-IR* ^*V2889*^/+; *rpr*-LacZ/+ (O) *Sd-*Gal4/*+*; *UAS-pll-IR* ^*V2889*^/+; *rpr*-LacZ/*UAS*-GFP (P) *Sd-*Gal4/*+*; *UAS-pll-IR* ^*V2889*^/+; *rpr*-LacZ/*UAS*-Pll (U) *Omb-*Gal4/*+* (V) *Omb-*Gal4/*+*; *UAS-pll-IR* ^*V2889*^/+ (W) *Omb-*Gal4/*+*; *UAS-pll-IR* ^*V2889*^/+; *UAS*-GFP/+ (X) *Omb-*Gal4/*+*; *UAS-pll-IR* ^*V2889*^/+; *UAS*-Pll/+.

**Fig 5:**
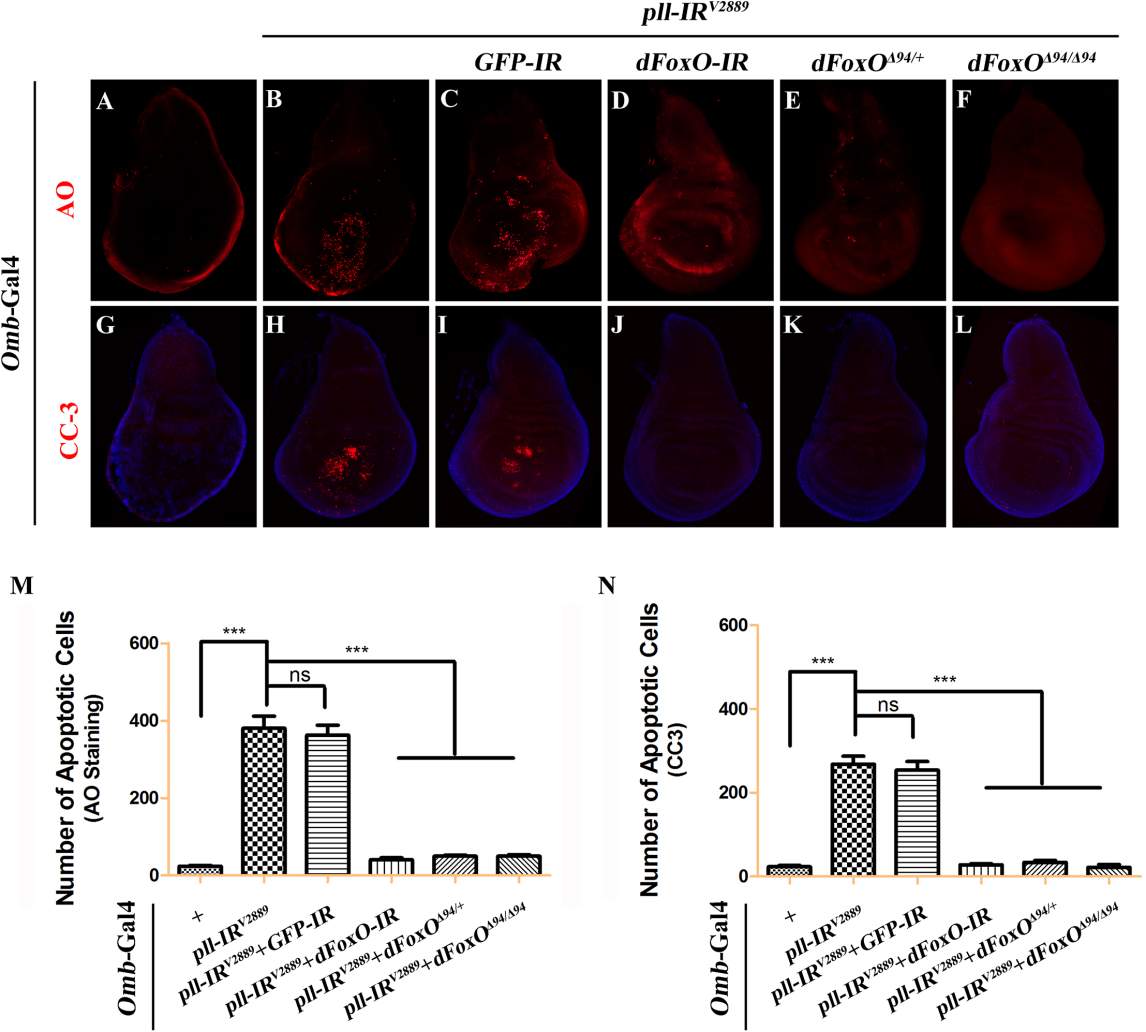
Loss-of-*pll* induces dFoxO-dependent cell death. Fluorescence micrographs of third instar larval wing discs stained with AO (**A-F**) or anti-Cleaved Caspase-3 (CC-3) antibody (**G-L**), anterior is to the left and dorsal up. Compared with controls (**A** and **G**), loss of *pll* resulted in increased cell death (**B**) and caspase activity (**H**), both of which were suppressed by knocking-down *dFoxO* (**D** and **J**), or deleting one or both copies of endogenous *dFoxO* (**E**, **F**, **K** and **L**). *GFP-IR* was used here as a negative control (**C** and **I**). (**M** and **N**) Quantifications of cell death by AO (**M**) and CC-3 antibody (**N**) staining as shown in figures **A-F** and **G-L** respectively. One-way ANOVA with Bonferroni multiple comparison test was used to compute *P*-values, significance is indicated with asterisks (*** *P*<0.001). ns stands for not significant. Detailed genotypes: (A and G) *Omb-*Gal4/*+* (B and H) *Omb-*Gal4/*+*; *UAS-pll-IR* ^*V2889*^/+ (C and I) *Omb-*Gal4/*+*; *UAS-pll-IR* ^*V2889*^/+; *UAS-GFP-IR*/+ (D and J) *Omb-*Gal4/*+*; *UAS-pll-IR* ^*V2889*^/+; *UAS-dFoxO-IR*/+ (E and K) *Omb-*Gal4/*+*; *UAS-pll-IR* ^*V2889*^/+; *dFoxO* ^Δ94/+^ (F and L) *Omb-*Gal4/*+*; *UAS-pll-IR* ^*V2889*^/+; *dFoxO* ^Δ94/Δ94^.

Apoptosis plays a crucial role in maintaining tissue homeostasis, making the decision between cell death and survival in response to various intracellular and extracellular stress [36,40]. To examine the role of *pll* in regulating cell death, we performed acridine orange (AO) staining and TdT-mediated dUTP nick end labelling (TUNEL) assay, both are commonly used to detect apoptosis [41,42]. We found that knock-down *pll* in the wing disc resulted in increased AO (Fig 4A, 4B, 4E and 4F; Fig 5A and 5B; S4A, S4B, S4K and S4L Fig) and TUNEL staining (Fig 4Q, 4R, 4U and 4V; S4G–S4J Fig) in the corresponding regions, which were soundly suppressed by the expression of Pll (Fig 4D, 4H, 4T and 4X), but not that of GFP (Fig 4C, 4G, 4S and 4W), indicating *pll* negatively regulates cell death in wing development.

**Fig 6:**
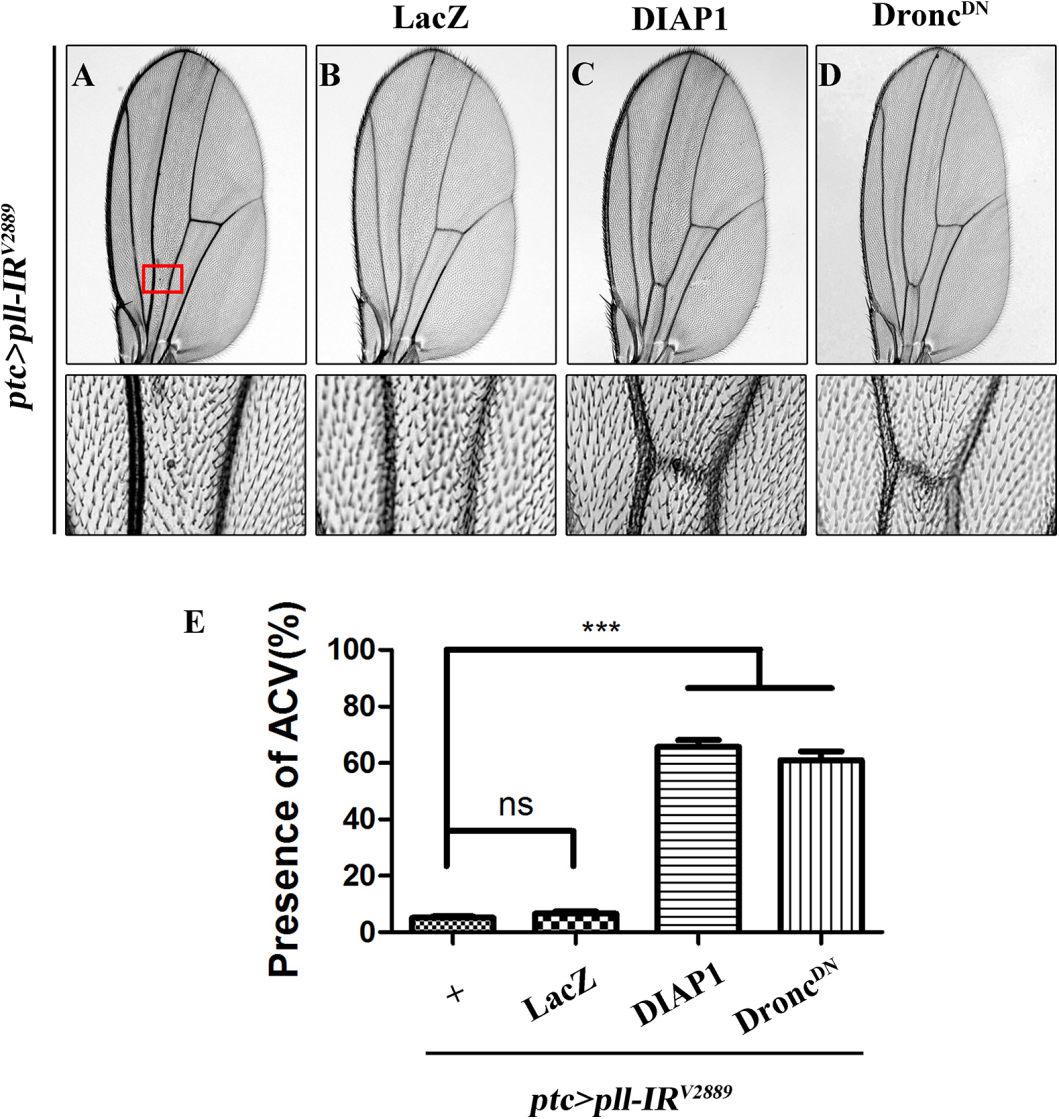
Depletion of *pll* elicits caspases-dependent cell death in adult wing. (**A-D**) Light micrographs showing *Drosophila* adult wings, anterior is to the left and distal up. The loss-of-ACV phenotype in *ptc>pll-IR* ^*V2889*^ flies (**A**) was significantly suppressed by the expression of DIAP1 (**C**) or Dronc^DN^ (**D**), but not that of LacZ (**B**), which served as a negative control. The lower panels are high magnification of the boxed areas in upper panels (**A-D**). (**E**) Quantification of the ACV phenotypes as shown in figures **A-D**. One-way ANOVA with Bonferroni multiple comparison test was used to compute *P*-values, significance is indicated with asterisks (*** *P*<0.001). ns stands for not significant. Detailed genotypes: (A) *ptc-*Gal4/*UAS-pll-IR* ^*V2889*^ (B) *ptc-*Gal4/*UAS-pll-IR* ^*V2889*^; *UAS*-LacZ/+ (C) *ptc-*Gal4/*UAS-pll-IR* ^*V2889*^; *UAS*-DIAP1/+ (D) *ptc-*Gal4/*UAS-pll-IR* ^*V2889*^; *UAS*-Dronc^DN^/+.

Apoptosis in *Drosophila* is triggered by transcriptional up-regulation of three pro-apoptotic genes *rpr*, *hid* and *grim*, and is mediated by the cleavage and activation of caspases [40]. Consistent with its role in cell death, loss of *pll* in the wing pouch results in up-regulated transcription of *hid* and *rpr* as revealed by X-gal staining of a *hid*-LacZ and an *rpr*-LacZ reporters (Fig 4I, 4J, 4M and 4N; S4C–S4F Fig), which is suppressed by expressing Pll, but not GFP (Fig 4K, 4L, 4O and 4P). Furthermore, we found that a ubiquitous knock-down of *pll* (*act>pll-IR*) was able to activate the transcription of endogenous *hid*, *rpr* and *grim*, as compared to the *act*-Gal4 control, via executing a real-time qRT-PCR assay (S4M Fig). In addition, depletion of *pll* leads to enhanced antibody staining for the activated form of Caspase-3 (CC-3, Fig 5G and 5H), a read-out of the initiator caspase Dronc activity [43]. Moreover, we found that the loss-of-ACV phenotype induced by *ptc>pll-IR* was suppressed partially by the deficiency *Df(3L)H99* that deletes *rpr*, *hid* and *grim* (S5A–S5C Fig), and significantly by expressing the inhibitor of apoptosis protein DIAP1 or a dominant-negative form of Dronc (Dronc^DN^), but not LacZ (Fig 6). Accordingly, the phenotype of reduced distal-most area in *Omb*>*pll-IR* wing is considerably rescued by *Df(3L)H99* (S5D–S5F Fig). Thus, we conclude from these data that depletion of *pll* is sufficient to activate caspase-mediated cell death.

### dFoxO is required for loss-of-*pll* induced cell death

**Fig 7:**
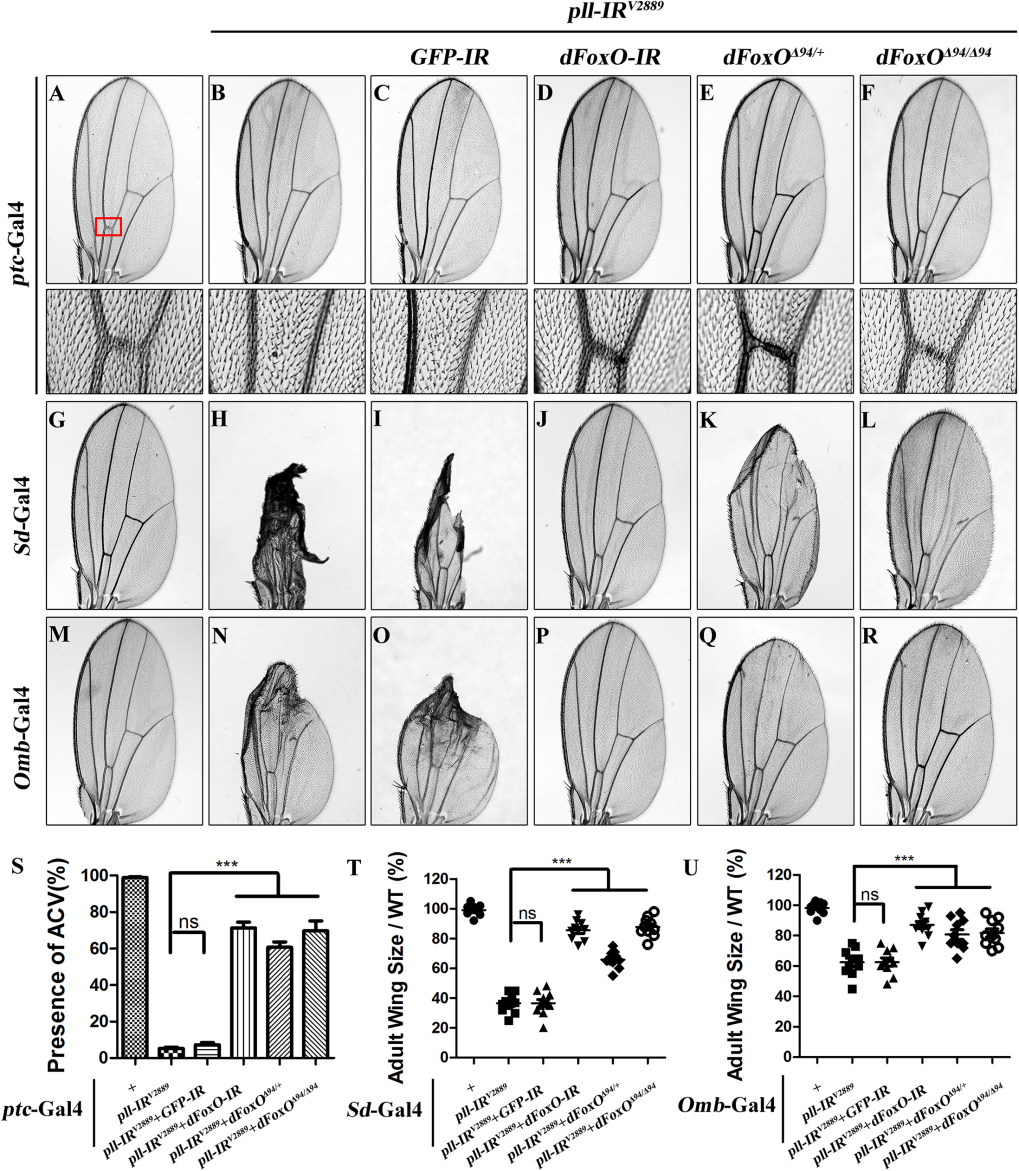
*dFoxO* is required for loss-of-*pll* induced wing phenotypes. (**A**-**R**) Light micrographs showing *Drosophila* adult wings, anterior is to the left and distal up. Compared with controls (**A**, **G** and **M**), the wing phenotypes of *ptc*>*pll-IR* ^*V2889*^ (**B**), *Sd>pll-IR* ^*V2889*^ (**H**) and *Omb>pll-IR* ^*V2889*^ (**N**) flies were suppressed by knocking-down *dFoxO* (**D**, **J** and **P**) or removing one or both copies of *dFoxO* (**E**, **F**, **K**, **L**, **Q** and **R**), but not by expressing a *GFP-IR* (**C**, **I** and **O**). In **A**-**F**, the lower panels are high magnification of the boxed areas in upper panels. (**S**-**U**) Statistical analysis of the ACV phenotype (**S**) and the adult wing size/wild type (WT) (**T** and **U**) as shown in figures **A**-**F**, **G**-**L** and **M**-**R** respectively. One-way ANOVA with Bonferroni multiple comparison test was used to compute *P*-values, significance is indicated with asterisks (*** *P*<0.001). ns stands for not significant. Detailed genotypes: (A) *ptc-*Gal4/*+* (B) *ptc-*Gal4/*UAS-pll-IR* ^*V2889*^ (C) *ptc*-Gal4/*UAS-pll-IR* ^*V2889*^/+; *UAS-GFP-IR*/+ (D) *ptc*-Gal4/*UAS-pll-IR* ^*V2889*^/+; *UAS-dFoxO-IR*/+ (E) *ptc*-Gal4/*UAS-pll-IR* ^*V2889*^/+; *dFoxO* ^Δ94/+^ (F) *ptc*-Gal4/*UAS-pll-IR* ^*V2889*^/+; *dFoxO* ^Δ94/Δ94^ (G) *Sd-*Gal4/*+* (H) *Sd-*Gal4/*+*; *UAS-pll-IR* ^*V2889*^/+ (I) *Sd-*Gal4/*+*; *UAS-pll-IR* ^*V2889*^/+; *UAS-GFP-IR*/+ (J) *Sd-*Gal4/*+*; *UAS-pll-IR* ^*V2889*^/+; *UAS-dFoxO-IR*/+ (K) *Sd-*Gal4/*+*; *UAS-pll-IR* ^*V2889*^/+; *dFoxO* ^Δ94/+^ (L) *Sd-*Gal4/*+*; *UAS-pll-IR* ^*V2889*^/+; *dFoxO* ^Δ94/Δ94^ (M) *Omb-*Gal4/*+* (N) *Omb-*Gal4/*+*; *UAS-pll-IR* ^*V2889*^/+ (O) *Omb-*Gal4/*+*; *UAS-pll-IR* ^*V2889*^/+; *UAS-GFP-IR*/+ (P) *Omb-*Gal4/*+*; *UAS-pll-IR* ^*V2889*^/+; *UAS-dFoxO-IR*/+ (Q) *Omb-*Gal4/*+*; *UAS-pll-IR* ^*V2889*^/+; *dFoxO* ^Δ94/+^ (R) *Omb-*Gal4/*+*; *UAS-pll-IR* ^*V2889*^/+; *dFoxO* ^Δ94/Δ94^.

To understand the mechanism by which loss of *pll* induces caspase-dependent cell death, we considered the transcription factor dFoxO as a putative downstream factor negatively regulated by Pll. Firstly, dFoxO is known to be negatively regulated by other kinase, e.g. Akt [26,28]. Secondly, previous studies reported that dFoxO is required for the apoptotic response and regulates the expression of pro-apoptotic gene *hid* [30]. Thirdly, we have previously observed a similar loss-of-ACV phenotype resulted from dFoxO expression driven by *ptc*-Gal4 driver [33]. In support of the assumption, we found that loss-of-*pll* triggered cell death in the wing pouch, as detected by AO staining (Fig 5B and 5M) and cleaved caspase-3 (CC-3) antibody (Fig 5H and 5N), was notably blocked by RNAi-mediated depletion of *dFoxO*, and in heterozygous or homozygous *dFoxO* ^***Δ94***^ mutants (Fig 5D–5F and 5J–5N). Furthermore, depletion of *pll* induced various wing defects are suppressed by loss of *dFoxO*, either by mutation or expression of RNAi (Fig 7; S6 Fig). A *GFP* RNAi was employed as a negative control (Fig 5C and 5I; Fig 7C, 7I and 7O). The knock-down efficiency of *dFoxO* RNAi lines and the *dFoxO* mRNA level in *dFoxO* ^***Δ94***^ mutants have been previously verified [33,44,45]. Consistently, expression of dFoxO driven by *ptc*-Gal4 (*ptc*>dFoxO) recapitulates the loss-of-ACV phenotype in *ptc*>*pll-IR* flies (S7A Fig). However, the *ptc*>dFoxO triggered loss-of-ACV phenotype cannot be suppressed by co-expressing Pll (S7B and S7C Fig), confirming that dFoxO acts downstream of Pll. Therefore, we conclude that loss of *pll* induces dFoxO-dependent cell death in wing development.

**Fig 8:**
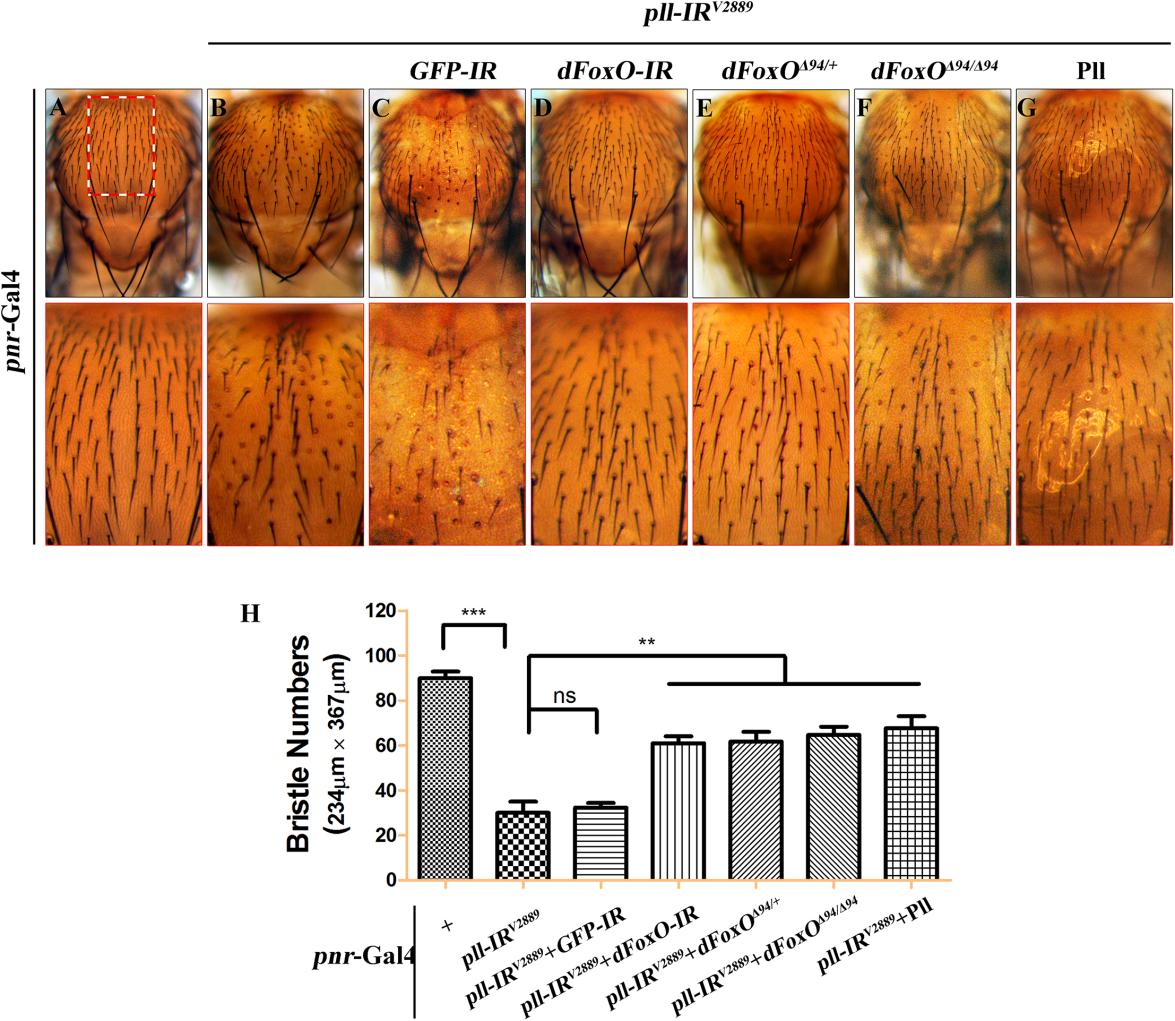
*dFoxO* is required for loss-of-*pll* induced bristle phenotype on notum. (**A-G**) Light micrographs showing bristles on the adult notum. Compared with the *pnr*-Gal4 control (**A**), RNAi-mediated depletion of *pll* in notum resulted in reduced bristle number (**B**), which was suppressed by knocking-down *dFoxO* (**D**), deleting one or both copies of endogenous *dFoxO* (**E** and **F**), or expressing Pll (**G**), but not by expressing a *GFP* RNAi that served as a negative control (**C**). The lower panels show high magnification view of the boxed areas in upper panels. (**H**) Quantification of bristles number in the boxed areas from **A-G**. One-way ANOVA with Bonferroni multiple comparison test was used to compute *P*-values, significance is indicated with asterisks (*** *P*<0.001, ** *P*<0.01). ns stands for not significant. Detailed genotypes: (A) *pnr-*Gal4/*+* (B) *UAS-pll-IR* ^*V2889*^/+; *pnr-*Gal4/*+* (C) *UAS-pll-IR* ^*V2889*^/+; *pnr-*Gal4/*UAS-GFP-IR* (D) *UAS-pll-IR* ^*V2889*^/+; *pnr-*Gal4/*UAS-dFoxO-IR* (E) *UAS-pll-IR* ^*V2889*^/+; *pnr-*Gal4/*dFoxO* ^***Δ94***^ (F) *UAS-pll-IR* ^*V2889*^/+; *pnr-*Gal4 *dFoxO* ^***Δ94***^/*dFoxO* ^***Δ94***^ (G) *UAS-pll-IR* ^*V2889*^/+; *pnr-*Gal4/*UAS*-Pll.

*pll* is ubiquitously expressed throughout development [6]. To investigate whether Pll modulates dFoxO-dependent cell death in other tissues, we knocked down *pll* in the notum by the *pnr*-Gal4 driver, and observed a loss-of-bristle phenotype in the notum (Fig 8A, 8B and 8H). This phenotype, most likely caused by cell death as expression of the apoptotic gene *reaper* in the notum produces a similar bristle-ablation phenotype [46], is significantly suppressed by expressing a *dFoxO* RNAi, or deleting one or both copies of endogenous *dFoxO*, or expressing Pll, but not a *GFP* RNAi (Fig 8C–8H). Thus, Pll negatively regulates dFoxO-dependent cell death in a non-tissue-specific manner.

### Pll regulates dFoxO subcellular localization and transcriptional activity

**Fig 9:**
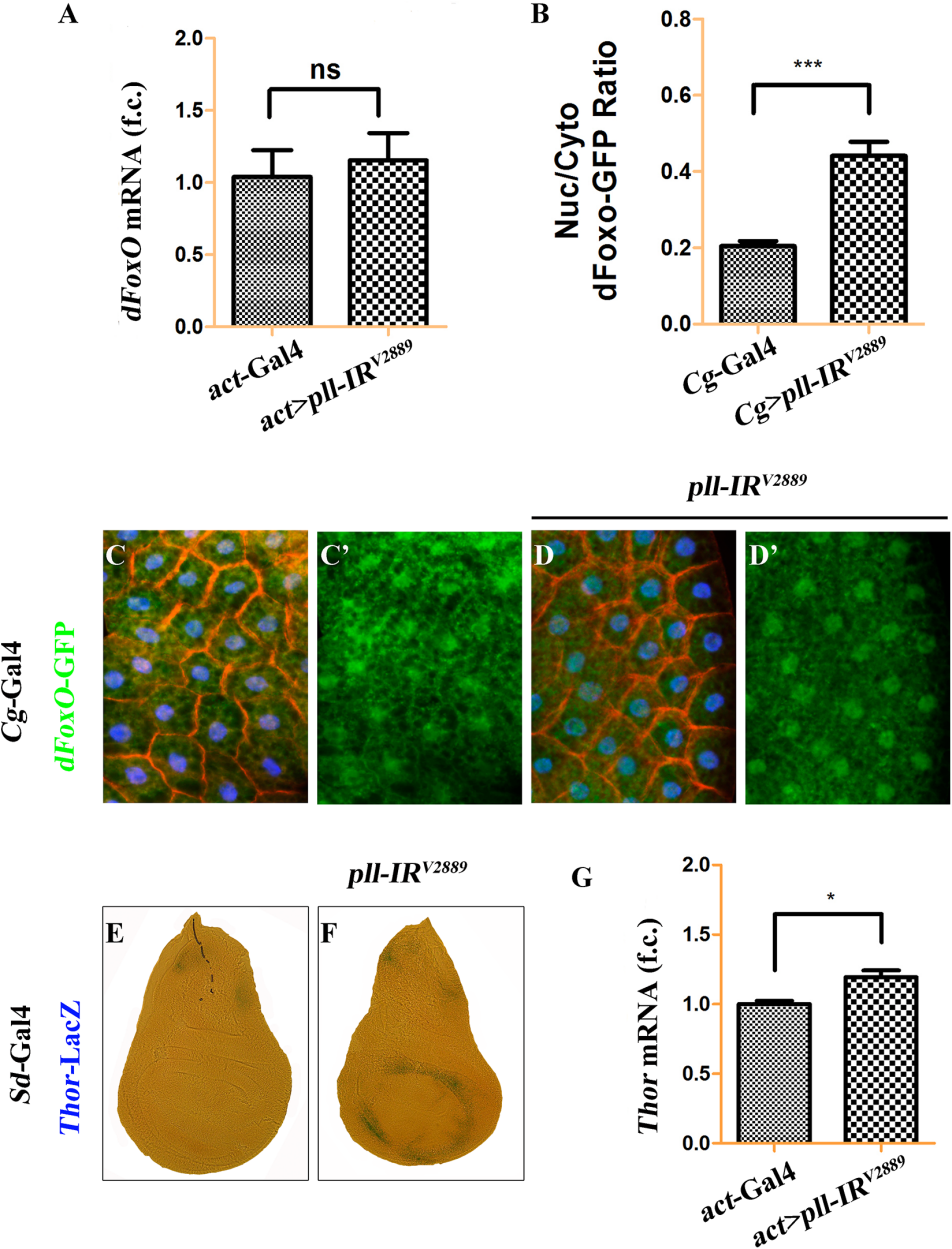
Loss of *pll* promotes dFoxO nuclear localization and transcriptional activity. (**A**) Loss of *pll* does not affect the transcription of *dFoxO*. Histogram showing the levels of *dFoxO* mRNAs measured by quantitative RT-PCR. Total RNA of *Drosophila* third instar larvae were extracted and normalized for cDNA synthesis. Error bars represents standard deviation from three independent experiments. ns stands for not significant. (**B-D**) Loss of *pll* promotes nuclear localization of dFoxO. (**B**) Quantification of the nuclear/cytoplasmic ratio of *dFoxO*-GFP fusion protein in the fat body shown in **C** and **D**. *dFoxO*-GFP intensities were measured in pixels using Image J. Error bars showed standard deviation from measurement of at least 15 cells for each genotype. Unpaired t test was used to calculate statistical significance, indicated with asterisks (*** *P*<0.001). (**C** and **D**) Fluorescence micrographs of fat body cells are shown. Compared with the control (**C**), loss of *pll* promotes the translocation of *dFoxO*-GFP from cytoplasm to nucleus (**D**). Nuclei were marked with DAPI (blue), cell membranes were stained by anti-Dlg antibody (red). (**E** and **F**) X-Gal staining of a *Thor*-LacZ reporter in third instar larval wing discs. Compared with the control (**E**), knock-down *pll* in the wing pouch induced *Thor* transcription (**F**). (**G**) Loss of *pll* up-regulated the level of *Thor* mRNA, as measured by quantitative RT-PCR. Total RNA of *Drosophila* third instar larvae were extracted and normalized for cDNA synthesis. Error bars represented standard deviation from three independent experiments. Unpaired t test was used to calculate statistical significance, indicated with asterisks (* *P*<0.05). Detailed genotypes: (A) Left: *act*-Gal4/+, Right: *UAS-pll-IR* ^*V2889*^/+; *act*-Gal4/+ (B) Left: *Cg*-Gal4/+, Right: *Cg-*Gal4/*UAS-pll-IR* ^*V2889*^ (C) *Cg-*Gal4/*+*; *dFoxO*-GFP/+ (D) *Cg-*Gal4/*UAS-pll-IR* ^*V2889*^; *dFoxO*-GFP/+ (E) *Sd*-Gal4/+; *Thor*-LacZ/+ (F) *Sd*-Gal4/+; *Thor*-LacZ/*UAS-pll-IR* ^*V2889*^ (G) Left: *act*-Gal4/+, Right: *UAS-pll-IR* ^*V2889*^/+; *act*-Gal4/+.

To address how Pll modulates dFoxO activity, we first checked whether Pll regulates dFoxO transcription by executing a real-time qRT-PCR assay. We found that a ubiquitous knockdown of *pll* (*act>pll-IR*) did not affect the mRNA level of *dFoxO*, as compared to the *act*-Gal4 control (Fig 9A), suggesting that *pll* does not regulate dFoxO transcription. Previous studies indicate that the nuclear localization of dFoxO is regulated by a series of post-translational modifications, including phosphorylation by different kinases [34]. To examine whether Pll modulates the nuclear-cytoplasmic shuttling of dFoxO, we checked the sub-cellular localization of a *dFoxO*-GFP fusion protein in the fat body. We found that the nuclear localization of *dFoxO*-GFP was significantly increased when *pll* was knocked down in fat body cells by *Cg*-Gal4 (Fig 9B–9D), which drives Gal4 expression specifically in fat body and hemocytes under the control of collagen (*Cg25C*) promoter [47]. To monitor the dFoxO activity directly, we detected the expression of its well-characterized target gene *Thor/4E-BP* by a *Thor-*LacZ reporter [26,28,48,49]. We found that *Thor-*LacZ expression was distinctly induced in the wing pouch by RNAi inactivation of *pll* under the control of *Sd*-Gal4 (Fig 9E and 9F). In addition, qRT-PCR assay confirmed that depletion of *pll* resulted in up-regulated *Thor/4E-BP* transcription (Fig 9G). Thus, we conclude that Pll regulates dFoxO subcellular localization and transcriptional activity.

### Pll physically interacts with and phosphorylates dFoxO

**Fig 10:**
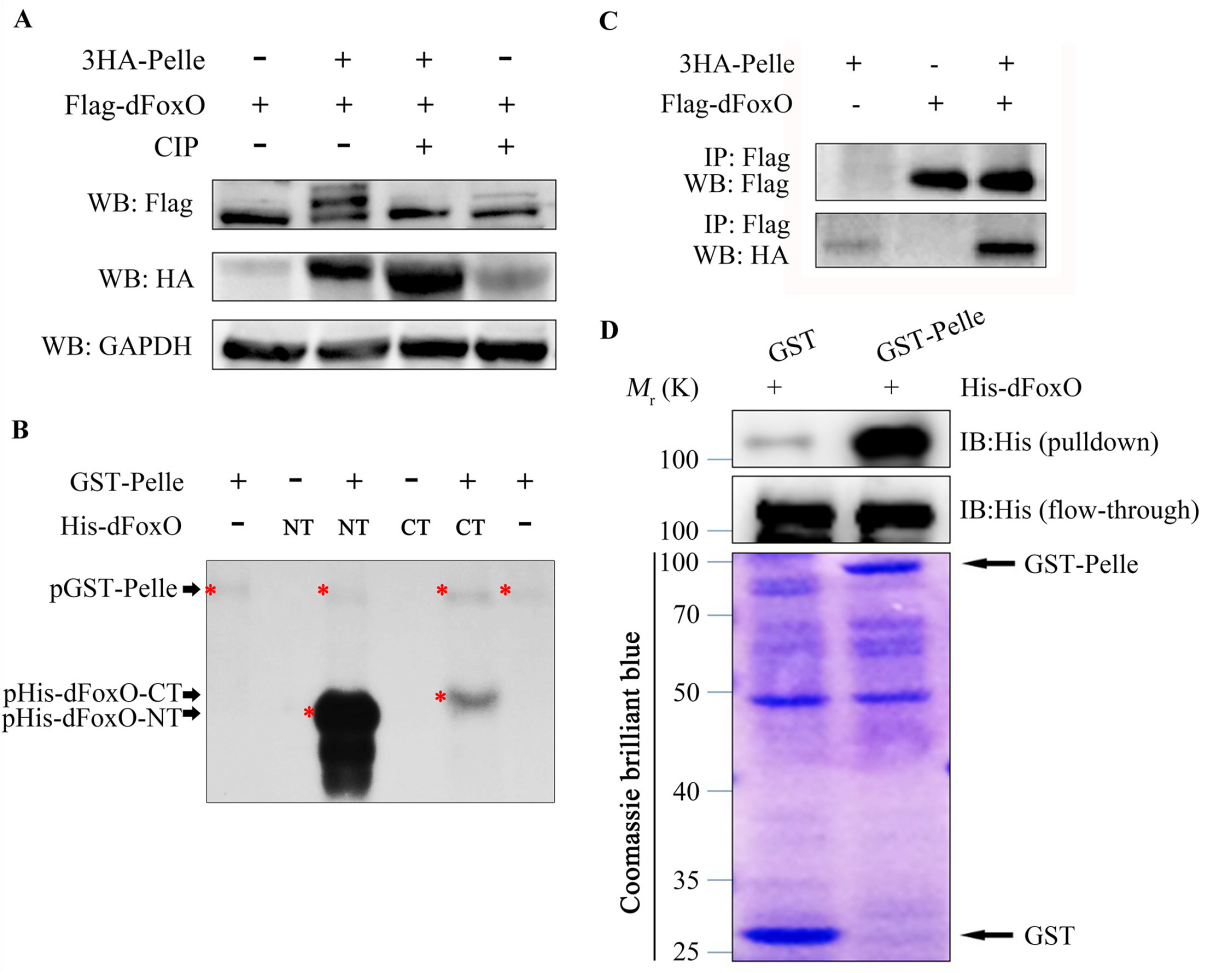
Pll binds to and phosphorylates dFoxO. (**A**) Expression of Pll induced mobility shift of dFoxO, which was abolished by CIP treatment. Cell lysis was prepared from S2R+ cells expressing Flag*-*dFoxO alone or with HA*-*Pelle, treated with or without calf intestine phosphatase (CIP), followed by western blotting. (**B)** Kinase assay shows Pll phosphorylates the N- and C- terminal parts of dFoxO. (**C**) Co-immunoprecipitation experiment shows dFoxO interacts with Pelle in S2R+ cells. (**D**) GST pull-down assay shows direct binding between bacterially expressed His–dFoxO and GST–Pll *in vitro*.

To investigate the underlying mechanism by which Pll regulates dFoxO activity, we expressed HA-tagged Pll (HA-Pll) and Flag-tagged dFoxO (Flag-dFoxO) in *Drosophila* S2R+ cells, and examined the phosphorylation of dFoxO through Calf Intestine Phosphatase (CIP) assay. We found that co-expression of Pll resulted in a mobility shift of dFoxO, which was abolished upon CIP treatment (Fig 10A), suggesting that dFoxO could be phosphorylated by Pll. Next, we performed the *in vitro* kinase assay to confirm the phosphorylation of dFoxO by Pll. Since Pll could be auto-phosphorylated (Fig 10B), to better distinguish the phosphorylated dFoxO from auto-phosphorylated Pll, we divided the full-length dFoxO (1-622aa) into two segments: the N-terminal (NT, 1-304aa) and the C-terminal (CT, 305-622aa). Indeed, bacterially purified GST-Pll was able to phosphorylate dFoxO *in vitro*, heavily on the NT and lightly on the CT (Fig 10B), suggesting Pll might phosphorylate dFoxO at multiple sites, most of which are located in the N-terminal half of dFoxO. Furthermore, Co-IP assay demonstrated a physical interaction between HA-Pll and Flag-dFoxO in S2R+ cells (Fig 10C). Finally, we performed the GST-pulldown assay and confirmed a direct binding between Pll and dFoxO (Fig 10D). Together, these data suggest that Pll could phosphorylate dFoxO through direct interaction, thus negatively regulates dFoxO activity by preventing its nuclear translocation.

## Discussion

*Drosophila melanogaster* has emerged as an excellent model organism to study apoptotic cell death and has made significant contribution to understand cell death regulation and its role in development. While mammalian IRAKs function as the mediator of IL-1Rs/TLRs signal transduction in the immune and inflammatory responses [1,2], Pll, the sole *Drosophila* orthologue of IRAKs, has been implicated as a central regulator of Toll pathway involved in embryonic dorsal/ventral patterning, innate immune response, muscle development and axon guidance. In this work, we identified a Toll pathway independent function of Pll in modulating caspase-mediated cell death in animal development.

Previous studies have suggested that *Drosophila* wing vein formation is a result of cell fate specification regulated by multiple signaling pathways including Notch, Hedgehog, EGF (epidermal growth factor) and BMP (bone morphogenetic proteins) pathways [50,51]. In the present study, we found that knock-down *pll* along the A/P compartment boundary of the developing wing (*ptc>pll-IR*) resulted in extensive cell death in the wing disc (Fig 4B) and a loss-of-ACV phenotype in the adult wing (Fig 1C; S1A and S1F Fig), implying a potential role of cell death in vein patterning. Consistent with this notion, the loss-of-ACV phenotype is rescued by blocking apoptotic cell death (Fig 6C–6E; S5B and S5C Fig), suggesting cell death is responsible for the loss of ACV. To investigate whether cell death is able to impede vein patterning, we initiated apoptosis by expressing the pro-apoptotic protein Grim under the control of *ptc*-Gal4. *ptc*>Grim caused extensive cell death in tissue ablation between L3 and L4 in the adult wing. In most cases, L3 and L4 were fused in the proximal area where ACV is located (S8A Fig). To adjust Grim expression and cell death, we added *Tub*-Gal80^ts^ that represses Gal4 activity in a temperature sensitive manner. At 25°C, *Tub*-Gal80^ts^ partially blocks *ptc-*Gal4 activity and allows limited Grim expression and therefore, cell death, between L3 and L4. Intriguingly, under this condition, we observed the loss-of-ACV phenotype accompanied by a slight reduction of area between L3 and L4 (S8B Fig), suggesting that both reduced area and loss-of-ACV phenotypes are consequences of cell death. From our experience, the loss-of-ACV phenotype is more sensitive to cell death, since weak cell death is sufficient to generate the phenotype, whereas stronger cell death is required to delete tissue between L3 and L4. Consistent with this notion, while *ptc*>*pll-IR* flies reared at 25°C only displayed the loss-of-ACV phenotype (Fig 1C), those raised at 29°C also showed reduced area between L3 and L4 (S8D and S8G Fig), which was caused by a reduction in cell number, but not cell size (S8E and S8F Fig).

We show that Pll regulates caspase activation and cell death through dFoxO. Mechanistically, loss of *pll* promotes the nuclear translocation of dFoxO, which otherwise is retained in the cytoplasm by phosphorylation. A number of kinases, including AKT, IκK and JNK, have been reported to phosphorylate FoxO and regulate its nuclear-cytoplasmic trafficking [52–54]. Here we provide evidence that Pll is another dFoxO kinase that phosphorylates dFoxO and inhibits its nuclear localization. Thus, it would be very interesting to check whether a similar interaction is conserved between IRAKs and FoxOs in mammal.

The FoxO family proteins have been implicated in multiple important biological processes, including cell death and tumor suppression. It has been reported that conditional deletion of FoxO1, FoxO3 and FoxO4 simultaneously results in the development of hemangiomas and thymic lymphomas [55], and IκB kinase represses FoxO3a activity to promote human breast tumorigenesis and acute myeloid leukemia (AML) [52,53]. IRAKs also show altered expression level in tumors and surrounding stroma [43,56], and participate in tumor initiation and progression [57], yet the underlying mechanisms remain poorly understood. Thus, the inhibitory effect of Pll on dFoxO activity in *Drosophila* provides a beneficial framework for a better understanding of mammalian IRAKs’ crucial roles in tumor development.

As the Toll/NF**-**κB pathway is not implicated in loss-of-*pll* triggered dFoxO-dependent cell death, we are curious about what are the pathways or factors act upstream of Pll to regulate its role in cell death? Since dFoxO has been reported as a downstream transcription factor in the JNK and Insulin pathways in *Drosophila* (S9P Fig; S10O Fig) [58–61], we wondered whether Pll is also involved in these pathways. Activation of JNK signaling between L3 and L4 by expressing Egr (*Drosophila* TNF) or Hep (*Drosophila* JNK Kinase), or depleting *puc* (encoding a JNK inhibitor), produced similar loss-of-ACV and reduced area phenotypes as that of *pll* depletion, yet the phenotypes were not affected by gain or loss of *pll* (S9 Fig). Inactivation of the Insulin pathway by expressing a dominant negative form of PI3K, or knocking-down *PI3K* or *Akt*, resulted in diminished area between L3 and L4, which remained unaffected by gain or loss of *pll* (S10 Fig). We also examined the Hippo pathway known to play a crucial role in regulating cell death and organ size [62]. Up-regulation of Hippo pathway in distinct wing areas by different Gal4 drivers led to various small wing or wing tissue ablation phenotypes, which were not altered by changing Pll level (S11 Fig). Finally, we checked dMyc, the fly homolog of c-Myc that regulates cell growth and cell death in *Drosophila* [63–65], and the cell polarity gene *scribble* (*scrib*), whose depletion promotes cell death [66–69]. We found that depletion of *dMyc* triggered wing phenotype and loss of *scrib* induced cell death are both independent of Pll (S12 Fig). Thus, while Pll directly regulates dFoxO-mediated caspase-dependent cell death in development, the upstream factors modulating Pll activity remain unknown, which deserve further investigation.

## Materials and Methods

### Fly strains

All *Drosophila* stocks were raised on a standard cornmeal and agar media, and crosses were performed at 25°C according to standard protocols unless otherwise indicated. *Drosophila* strains used include: *ptc*-Gal4, *Omb*-Gal4, *en*-Gal4, *act-*Gal4, *nub*-Gal4, *Cg*-Gal4 (7011), *Tub*-Gal80^ts^ (7017), *UAS*-RFP, *UAS*-LacZ, *UAS*-Dcr2, *UAS*-Toll^10B^, *UAS*-Cactus, *UAS*-DIAP1, *UAS*-dFoxO, *UAS*-Grim^M146^, *UAS*-Hep, *UAS-GFP-IR*, *UAS-Toll-IR* (31044, 31447 and 35628), *UAS-dorsal-IR* (27650, 32934 and 34938), *UAS-Dif-IR* (29514 and 30513), *UAS-dFoxO-IR*, *UAS-puc-IR* (3018), *UAS-PI3K-IR* (27690), *Df(3L)H99*, *dFoxO* ^***Δ94***^, *pll* ^*2*^ (3111), *pll* ^*7*^ (3112), *dFoxO*-GFP (37585), *hid*-LacZ, *Thor*-LacZ (9558) and *y w hs*-Flp; *act*>y+>Gal4 *UAS*-GFP were obtained from Bloomington stock center, *UAS-pelle-IR* (2889 and 103774), *UAS-dorsal-IR* (45996 and 45998), *UAS-Dif-IR* (30578 and 30579), *UAS-yki-IR* (40497), *UAS-dMyc-IR* (2948) and *UAS-scrib-IR* (27424) were obtained from Vienna *Drosophila* RNAi Center (VDRC), *UAS-tube-IR* (10520R1 and 10520R3), *UAS-dorsal-IR* (6667R2 and 6667R5), *UAS-cactus-IR* (5848R3) and *UAS-Akt-IR* (4006R1) were obtained from Japanese National Institute of Genetics (NIG), *UAS*-Pelle (201696) was obtained from *Drosophila* Genetic Resource Center (DGRC). *GMR*-Gal4 [70], *pnr-*Gal4 [71–73], *Sd*-Gal4 [71–73], *UAS-pelle-IR* ^*W*^ [74], *UAS*-Dronc^DN^ [46], *UAS*-GFP [71–73], *reaper*-LacZ [75], *UAS*-Egr^W^ [46], *UAS*-PI3K^DN^ [76], *UAS*-Warts [77] and *UAS*-Hippo [78] were previously described.

### Immunohistochemistry

Imaginal wing discs dissected from third instar larvae were collected in cold PBS and fixed in 4% paraformaldehyde. After proper washes, the wing discs were blocked in 10% horse serum, and stained with antibodies. The following antibodies were used: rabbit anti-pH3 (1:200 Cell Signaling Technology), rabbit anti-Cleaved Caspase-3 (1:200 Cell Signaling Technology) and mouse anti-Dlg (1:200). Secondary antibodies were anti-rabbit-Alexa (1:1000 Cell Signaling Technology) and anti-mouse-Cy3 (1:1000, Jackson Immuno Research).

### AO staining

Wing discs were dissected from third instar larvae in PBST and incubated in 1×10^−5^ M AO for 5 min at room temperature prior to imaging as described [79].

### X-Gal staining

Wing discs were dissected from third instar larvae in PBST (1×PBS pH 7.0, 0.1% Triton X-100) and stained for ß-galactosidase activity as described [80].

### TUNEL staining

The wing discs of wandering third-instar were dissected out in PBS. Wing discs were fixed in 4% Paraformaldehyde for 30 min at room temperature (RT) and washed with PBS-Tx (0.3% Triton100) three times for 30 min. TUNEL staining was performed using the Fluorescein Cell Death Kit produced by Boster Company. Imaging of prepared sample was conducted by a Leica confocal microscope (Leica SP5).

### qRT-PCR

Five third instar larvae of indicated genotypes were collected and total RNA was isolated using TRIzol (Invitrogen). qRT-PCR was performed as previously described [81] using following primers:

For *rp49* Sense: 5’- TACAGGCCCAAGATCGTGAA-3’

            Antisense: 5’- TCTCCTTGCGCTTCTTGGA-3’

For *pelle* Sense: 5’GTGGTAAGCCGTGCCTCGTCTA-3’

            Antisense: 5’-CTGCCAGGTGAGTGCTGGTAGT-3’

For *dFoxO* Sense: 5’- CAATCTCGAGTGCAATGTCGAGGA -3’

            Antisense: 5’-CCCTGAGCATCAGCAACATTAGCA-3’

For *Thor* Sense: 5’-TCGGAGTTTGGATGCTGGGATCTT-3’

            Antisense: 5’-AGTCACGTCGTCCTCATCGTTGTT-3’

For *hid* Sense: 5’-TGCGAAATACACGGGTTCA-3’

            Antisense: 5’-CCAATATCACCCAGTCCCG-3’

For *rpr* Sense: 5’-GAGCAGAAGGAGCAGACGAT-3’

            Antisense: 5’-CGATATTTGCCGGACTTTCT-3’

For *grim* Sense: 5’-TCGGAGTTTGGATGCTGGGATCTT-3’

            Antisense: 5’-AGTCACGTCGTCCTCATCGTTGTT-3’

### Western blotting, co-immunoprecipitation and phosphatase treatment

*Drosophila* S2R+ cells were cultured in Corning Insectagro DS2 with 10% FBS (HyClone). Plasmids pUAST-Flag-dFoxO, pUAST-3HA-Pelle and Actin-GAL4 were used for co-transfection as indicated using Effectene Transfection Reagent (QIAGEN). 48 hours after transfection, cells were lysed in RIPA buffer (CST) with PMSF and proceeded with the standard western blot and co-immunoprecipitation protocols. Proteins were probed or immunoprecipitated with following antibodies: Flag-Tag antibody [3A6] (CMCTAG), HA-Tag (C29F4) Rabbit mAb (CST), Anti dFoxO (Cosmo Bio), Anti-rabbit IgG, HRP-linked Antibody (CST), Anti-mouse IgG, HRP-linked Antibody (CST). For Phosphatase Treatment, cell extracts were incubated with calf intestine phosphatase (CIP) (New England BioLabs) for 40 mins at 37°C.

### *In vitro* kinase assays

The GST-Pelle plasmid is a gift from Dr. Wasserman at UCSD. GST-Pelle protein was induced to express in BL21 cells and purified as described [37] except that cells were lysed by sonication. PCR amplified DNA fragments of dFoxO were constructed into pET.M.3C vector (gift from Dr. Long at Nankai University) for expressing proteins of full-length (1-622aa), N-terminal (1-304aa) and C-terminal (305-622aa) dFoxO, respectively. Each resulting protein contains 6ᵡHis-tag at N termini designed as His-dFoxO, His-dFoxONT or His-dFoxOCT. BL21 cells transformed with the constructs were grown for 15 hours at 16°C following 0.8mM IPTG induction, and 6ᵡHis-tagged proteins were purified as previously described [82].

The *in vitro* kinase assays were performed as described [37]. Briefly, GST-Pelle (30 μg) was pre-incubated for 45 mins at 30°C in 1×kinase buffer (10mM ATP, 10mM MgCl_2_, 50mM β-glycerophosphate, 25mM HEPES, pH6.5) to allow activation by auto-phosphorylation. The activated Pelle (6 μg) was then incubated with recombinant 6ᵡHis-tagged dFoxO/NT/CT (10 μg) in a volume of 40 μl in the presence of [γ-32P]-ATP (5 μ Curie, Perkin Elmer) in 1×kinase buffer. Following reaction for 5 mins at 30°C, samples were mixed with 10 μl 5×SDS loading sample buffer, boiled, and loaded (25 ml) on an 10% SDS protein gel.

### GST-pulldown assays

The GST-pulldown assays were performed as previous described [83]. Recombinant GST-Pelle-λPPase and 6ᵡHis-dFoxo proteins were produced in BL21 cells and purified with glutathione-Sepharose 4B (GE Healthcare) or Ni-NTA resin (GE Healthcare) respectively according to standard protocols. 2 μg GST or 10 μg GST-fusion proteins was incubated at 4°C for 5 hours with 10 μg purified 6ᵡHis-dFoxo and 50 μl of glutathione-Sepharose beads. Supernatants were collected as input and the Sepharose beads were then extensively washed 5 times with lysis buffer and resuspended in SDS loading buffer and boiled. A quarter of the sample buffer was loaded in 12% SDS-PAGE for detection with anti-His antibody (Sungene Biotech).

## Supporting Information

S1 Fig Depletion of *pll* produces a loss-of-ACV phenotype in adult wings. (**A-G**) Light micrographs showing *Drosophila* adult wings. Expression of two additional *pll* RNAi, *pll-IR* ^*W*^ (**A**) and *pll-IR* ^*V103774*^ (**F**), driven by *ptc*-Gal4 generated a loss-of-ACV phenotype, which was rescued by expression of Pll (**G**). Heterozygous for two *pll* mutants, *pll* ^*2*^ (**D**) and *pll* ^*7*^ (**E**), showed no obvious defects, but strongly enhanced *ptc>pll-IR* ^*W*^ induced loss-of-ACV phenotype (**B** and **C**). The lower panels show high magnification view of the boxed areas in upper panels. (**H**) Statistical analysis of the ACV phenotype shown in figures **A**-**G**. One-way ANOVA with Bonferroni multiple comparison test was used to compute *P*-values, significance is indicated with asterisks (*** *P*<0.001). Detailed genotypes: (A) *ptc-*Gal4/*UAS-pll-IR* ^*W*^ (B) *ptc-*Gal4/*UAS-pll-IR* ^*W*^; *pll* ^*2*^/+ (C) *ptc-*Gal4/*UAS-pll-IR* ^*W*^; *pll* ^*7*^/+ (D) *ptc-*Gal4/*+*; *pll* ^*2*^/+ (E) *ptc-*Gal4/*+*; *pll* ^*7*^/+ (F) *ptc-*Gal4/*UAS-pll-IR* ^*V103774*^ (G) *ptc-*Gal4/*UAS-pll-IR* ^*V103774*^; *UAS*-Pll/+.
(TIF)

S2 Fig Verification of RNAi lines for *pll* and Toll pathway components. (**A-K**) Light micrographs of *Drosophila* adult eyes are shown. Compared with the *GMR-*Gal4 control (**A**), *GMR*>Pll produced a rough eye phenotype (**B**) that was significantly suppressed by three independent *pll* RNAi lines (**C-E**). The rough eye phenotype of *GMR*>Toll^10B^ (Tl^10B^) (**F**) was fully suppressed by RNAi down-regulation of Toll pathway components: *Toll*, *tube*, *dorsal* and *Dif*, or ectopic expression of Cactus (**G-K**). (**L**) The knock-down efficacies of *pll* RNAi lines. Expression of three *pll* RNAi, but not LacZ, significantly reduced the level of *pll* mRNA, as measured by quantitative RT-PCR. Total RNA of *Drosophila* third instar larvae were extracted and normalized for cDNA synthesis. Error bars represent standard deviation from three independent experiments. Parison test was used to compute *P*-values, significance is indicated with asterisks. ** *P*<0.01, ns stands for not significant. Detailed genotypes: (A) *GMR*-Gal4/+ (B) *GMR*-Gal4/*UAS*-Pll (C) *UAS-pll-IR* ^*V2889*^/+; *GMR*-Gal4/*UAS*-Pll (D) *UAS-pll-IRV* ^*103774*^/+; *GMR*-Gal4/*UAS*-Pll (E) *UAS-pll-IR* ^*W*^/+; *GMR*-Gal4/*UAS*-Pll (F) *UAS*-Toll^10B^/+; *GMR*-Gal4/*+* (G) *UAS*-Toll^10B^/+; *GMR*-Gal4/*UAS-Toll-IR* (H) *UAS*-Toll^10B^/+; *GMR*-Gal4/*UAS-tube-IR* (I) *UAS*-Toll^10B^/*UAS*-Cactus; *GMR*-Gal4/*+* (J) *UAS*-Toll^10B^/+; *GMR*-Gal4/*UAS-dorsal-IR* (K) *UAS*-Toll^10B^/+; *GMR*-Gal4/*UAS-Dif-IR*.
(TIF)

S3 Fig Loss of *pll* results in wing tissue ablation. (**A**-**C**, **E**-**G** and **I**-**K**) Light micrographs of *Drosophila* adult wings are shown. Compared with controls (**A**, **E** and **I**), expression of *pll* RNAi driven by *Omb*-Gal4 (**B**), *Sd*-Gal4 (**F**) or *en*-Gal4 (**J**) resulted in reduced wing sizes, which were suppressed by expressing Pll (**C**, **G** and **K**). Quantifications of adult wing size/wild type (WT) (**D** and **H**) and total size P/A ratio (**L**) are shown for indicated genotypes. One-way ANOVA with Bonferroni multiple comparison test was used to compute *P*-values, significance is indicated with asterisks (*** *P*<0.001). Detailed genotypes: (A) *Omb*-Gal4/+ (B) *Omb-*Gal4/+; *UAS-pll-IR* ^*V103774*^/+ (C) *Omb-*Gal4/+; *UAS-pll-IR* ^*V103774*^/+; *UAS*-Pll/+ (E) *Sd-*Gal4/*+* (F) *Sd-*Gal4/+; *UAS-pll-IR* ^*V103774*^/+ (G) *Sd-*Gal4/+; *UAS-pll-IR* ^*V103774*^/+; *UAS*-Pll/+ (I) *en-*Gal4/*+* (J) *en-*Gal4/*UAS-pll-IR* ^*V103774*^ (K) *en-*Gal4/*UAS-pll-IR* ^*V103774*^; *UAS*-Pll/+
(TIF)

S4 Fig Loss of *pll* elicits apoptotic cell death in wing discs. (**A**, **B**, **K** and **L**) AO staining of third instar larval wing discs. Compared with controls (**A** and **K**), knock down *pll* by *Sd*-Gal4 (**B**) or *Omb*-Gal4 (**L**) triggered cell death detected by AO staining. (**C**-**F**) X-Gal staining of a *hid*-LacZ and an *rpr*-LacZ reporters in wing discs. Compared with controls (**C** and **E**), knock down *pll* in the wing pouch induced *hid* (**D**) and *rpr* (**F**) transcription. (**G-J**) TUNEL staining of third instar larval wing discs. Compared with Gal4 controls (**G** and **I**), knock down *pll* by *Sd*-Gal4 (**H**) or *Omb*-Gal4 (**J**) induced cell death in the corresponding areas. In all figures, anterior is to the left and dorsal up. (**M**) Loss of *pll* up-regulated the mRNA level of *hid*, *rpr* and *grim*, as measured by quantitative RT-PCR. Total RNA of *Drosophila* third instar larvae were extracted and normalized for cDNA synthesis. One-way ANOVA with Bonferroni multiple comparison test was used to calculate statistical significance, indicated with asterisks (* *P*<0.05, ** *P*<0.01). Error bars represent standard deviation from three independent experiments. Detailed genotypes: (A and G) *Sd-*Gal4/*+* (B and H) *Sd-*Gal4/*+*; *UAS-pll-IR* ^*V103774*^/+ (C) *Sd-*Gal4/*+*; *hid*-LacZ/+ (D) *Sd-*Gal4/*+*; *UAS-pll-IR* ^*V103774*^/+; *hid*-LacZ/+ (E) *Sd-*Gal4/*+*; *rpr*-LacZ/+ (F) *Sd-*Gal4/*+*; *UAS-pll-IR* ^*V103774*^/+; *rpr*-LacZ/+ (I and K) *Omb-*Gal4/*+* (J and L) *Omb-*Gal4/*+*; *UAS-pll-IR* ^*V103774*^/+.
(TIF)

S5 Fig *Df(3L)H99* suppresses loss-of-*pll* triggered wing phenotypes. (**A**, **B**, **D** and **E**) Light micrographs showing *Drosophila* adult wings. Wing phenotypes of *ptc>pll-IR* ^*V2889*^ (**A**) and *Omb>pll-IR* ^*V2889*^ (**D**) were partially suppressed by *Df(3L)H99* that deletes one copy of the apoptotic genes *reaper*, *hid* and *grim* (**B** and **E**). The lower panels show high magnification view of the boxed areas in upper panels (**A** and **B**). Statistical analysis of the ACV phenotype (**C**) and quantification of adult wing size/WT (**F**) are shown for indicated genotypes. Unpaired t test was used to calculate statistical significance, indicated with asterisks (*** *P*<0.001). Detailed genotypes: (A) *ptc*-Gal4/*UAS-pll-IR* ^*V2889*^ (B) *ptc*-Gal4/*UAS-pll-IR* ^*V2889*^; *H99*/+ (D) *Omb*-Gal4/+; *UAS-pll-IR* ^*V2889*^/+ (E) *Omb*-Gal4/+; *UAS-pll-IR* ^*V2889*^/+; *H99*/+.
(TIF)

S6 Fig *dFoxO* is required for loss-of-*pll* induced wing phenotype. (**A**-**D**) Light micrographs showing *Drosophila* adult wings. The loss-of-ACV phenotype in *ptc*>*pll-IR* ^*V103774*^ flies (**A**) was suppressed by removing one copy of endogenous *dFoxO* (**C**) or expressing a *dFoxO* RNAi (**D**), but not by expressing LacZ (**B**). The lower panels show high magnification view of the boxed areas in upper panels. (**E**) Quantification of the ACV phenotype as shown in figures **A**-**D**. One-way ANOVA with Bonferroni multiple comparison test was used to compute *P*-values, significance is indicated with asterisks (*** *P*<0.001). Detailed genotypes: (A) *ptc-*Gal4/*UAS-pll-IR* ^*V103774*^ (B) *ptc-*Gal4/*UAS-pll-IR* ^*V103774*^; *UAS*-LacZ/+ (C) *ptc-*Gal4/*UAS-pll-IR* ^*V103774*^; *dFoxO* ^***Δ94***^/+ (D) *ptc-*Gal4/*UAS-pll-IR* ^*V103774*^; *UAS-dFoxO-IR*/+.
(TIF)

S7 Fig Expression of Pll does not suppress dFoxO-induced wing phenotype. (**A** and **B**) Light micrographs of *Drosophila* adult wings are shown. Expression of dFoxO driven by *ptc*-Gal4 recapitulated the loss-of-ACV phenotype (**A**), which was not suppressed by the expression of Pll (**B**). (**C**) Statistical analysis of the ACV phenotype shown in figures **A** and **B**. Unpaired t test was used to calculate statistical significance. ns stands for not significant. Detailed genotypes: (A) *ptc-*Gal4/*UAS-*dFoxO (B) *ptc-*Gal4/*UAS-*dFoxO; *UAS*-Pll/+.
(TIF)

S8 Fig Cell death phenotypes triggered by Grim expression or *pll* depletion. (**A**-**D**) Light micrographs showing *Drosophila* adult wings. Compared with the *ptc*-Gal4 control (**C**), expression of Grim triggered strong cell death that resulted in partially fused L3 and L4 (**A**), while limited Grim expression imposed by *Tub*-Gal80^ts^ produced a loss-of-ACV phenotype (**B**). Enhanced expression of a *pll* RNAi (at 29°C) not only abolished ACV, but also diminished the area between L3 and L4 (**D**). (**E**-**G**) Quantifications of cell size (**E**), cell number (**F**) and area size (**G**) of L3-L4/total ratio in **C** and **D** are shown. While cell size was not affected, both cell number and area size decreased significantly when *pll* was knocked down by *ptc*-Gal4. Unpaired t test was used to calculate statistical significance, indicated with asterisks (**P<0.01, n = 10 in each group). ns stands for not significant. Detailed genotypes: (A) *ptc-*Gal4/*UAS*-Grim (B) *ptc-*Gal4/*UAS*-Grim; *Tub-*Gal80^ts^/+ (C) *ptc-*Gal4/*+* (D) *ptc-*Gal4/*UAS-pll-IR* ^*V2889*^.
(TIF)

S9 Fig Pll is not involved in JNK pathway-triggered cell death. (**A**-**M**) Light micrographs of *Drosophila* adult wings are shown. Compared with *ptc*-Gal4 control (**A**), expression of Egr^w^ (**B**) or Hep (**F**), or RNAi-mediated down-regulation of *puc* (**J**) along the A/P compartment boundary resulted in a loss-of-ACV phenotype and size reduction between L3 and L4. The phenotypes are not affected by gain- or loss-of-*pll*, or by expression of LacZ (**C**-**E**, **G**-**I** and **K**-**M**). Statistics analysis of area size L3-L4/total ratio (**N**) and the ACV phenotype (**O**) are shown for figures **A**-**M**. One-way ANOVA with Bonferroni multiple comparison test was used to compute *P*-values, significance is indicated with asterisks (*** *P*<0.001). ns stands for not significant. (**P**) A diagram for the key components of JNK pathway. Detailed genotypes: (A) *ptc-*Gal4/*+* (B) *ptc-*Gal4/*UAS-*Egr^W^ (C) *ptc-*Gal4/*UAS-*Egr^W^; *UAS*-Pll/+ (D) *ptc-*Gal4/*UAS-*Egr^W^; *pll* ^*7*^/+ (E) *ptc-*Gal4/*UAS-*Egr^W^; *UAS-*LacZ/+ (F) *ptc-*Gal4/*UAS-*Hep (G) *ptc-*Gal4/*UAS-*Hep; *UAS*-Pll/+ (H) *ptc-*Gal4/*UAS-*Hep; *pll* ^*7*^/+ (I) *ptc-*Gal4/*UAS-*Hep; *UAS-*LacZ/+ (J) *ptc-*Gal4/*UAS-puc-IR* (K) *ptc-*Gal4/*UAS-puc-IR*; *UAS*-Pll/+ (L) *ptc-*Gal4/*UAS-puc-IR*; *pll* ^*7*^/+ (M) *ptc-*Gal4/*UAS-puc-IR*; *UAS-*LacZ/+.
(TIF)

S10 Fig Pll is not involved in the Insulin signaling. (**A**-**M**) Light micrographs showing *Drosophila* adult wings. Compared with *ptc*-Gal4 control (**A**), expression of a dominant negative form of PI3K (**B**), or RNAi-mediated inactivation of *PI3K* (**F**) or *Akt* (**J**) led to a size reduction between L3 and L4, which was not affected by changing Pll level or expressing LacZ (**C**-**E**, **G**-**I** and **K**-**M**). (**N**) Quantification of adult wing size L3-L4/total ratio as shown in figures **A**-**M.** One-way ANOVA with Bonferroni multiple comparison test was used to compute *P*-values, significance is indicated with asterisks (*** *P*<0.001). ns stands for not significant. (**O**) A diagram for the key components of Insulin pathway. Detailed genotypes: (A) *ptc-*Gal4/*+* (B) *ptc-*Gal4/+; *UAS-*PI3K^DN^/+ (C) *ptc-*Gal4/+; *UAS-*PI3K^DN^/*UAS*-Pll (D) *ptc-*Gal4/+; *UAS-*PI3K^DN^/*pll* ^*7*^ (E) *ptc-*Gal4/+; *UAS-*PI3K^DN^/*UAS-*LacZ (F) *ptc-*Gal4/+; *UAS-PI3K-IR*/+ (G) *ptc-*Gal4/+; *UAS-PI3K-IR*/*UAS*-Pll (H) *ptc-*Gal4/+; *UAS-PI3K-IR*/*pll* ^*7*^ (I) *ptc-*Gal4/+; *UAS-PI3K-IR*/*UAS-*LacZ (J) *ptc-*Gal4/*UAS-Akt-IR* (K) *ptc-*Gal4/*UAS-Akt-IR*; *UAS*-Pll/+ (L) *ptc-*Gal4/*UAS-Akt-IR*; *pll* ^*7*^/+ (M) *ptc-*Gal4/*UAS-Akt-IR*; *UAS-*LacZ/+.
(TIF)

S11 Fig Pll is not involved in the Hippo pathway. **(A**-**E, G**-**K, M**-**Q** and **S**-**W)** Light micrographs of *Drosophila* adult wings are shown. Compared with controls (**A**, **G**, **M** and **S**), up-regulated Hippo signaling by expressing Hippo (Hpo) under the control of *nub*-Gal4 or *Sd*-Gal4 (**B** and **H**), or expressing Warts by *Sd*-Gal4 (**N**), or knocking-down *yki* by *ptc*-Gal4 (**T**) resulted in reduced wing tissue in the corresponding areas, which were not altered by changing Pll level or expressing LacZ (**C**-**E**, **I**-**K**, **O**-**Q** and **U**-**W**). (**F**, **L**, **R** and **X**) Quantifications of total wing size/wild type (WT) ratio or wing size L3-L4/total ratio are shown for figures **A**-**E**, **G**-**K**, **M**-**Q** and **S**-**W** respectively (n = 10). One-way ANOVA with Bonferroni multiple comparison test was used to compute *P*-values, significance is indicated with asterisks (*** *P*<0.001). ns stands for not significant. (**Y**) A diagram for the key components of Hippo pathway. Detailed genotypes: (A) *nub-*Gal4/*+* (B) *nub-*Gal4/+; *UAS-*Hippo/+ (C) *nub-*Gal4/+; *UAS-*Hippo/*UAS*-Pll (D) *nub-*Gal4/+; *UAS-*Hippo/*pll* ^*7*^ (E) *nub-*Gal4/+; *UAS-*Hippo/*UAS-*LacZ (G) *Sd*-Gal4/+ (H) *Sd*-Gal4/+; *UAS-*Hippo/+ (I) *Sd*-Gal4/+; *UAS-*Hippo/*UAS*-Pll (J) *Sd*-Gal4/+; *UAS-*Hippo/*pll* ^*7*^ (K) *Sd*-Gal4/+; *UAS-*Hippo/*UAS-*LacZ (M) *Sd*-Gal4/+ (N) *Sd*-Gal4/+; *UAS-*Warts/+ (O) *Sd*-Gal4/+; *UAS-*Warts/*UAS*-Pll (P) *Sd*-Gal4/+; *UAS-*Warts/*pll* ^*7*^ (Q) *Sd*-Gal4/+; *UAS-*Warts/*UAS-*LacZ (S) *ptc-*Gal4/+ (T) *ptc-*Gal4/+; *UAS-yki-IR*/+ (U) *ptc-*Gal4/+; *UAS-yki-IR*/*UAS*-Pll (V) *ptc-*Gal4/+; *UAS-yki-IR*/*pll* ^*7*^ (W) *ptc-*Gal4/+; *UAS-yki-IR*/*UAS-*LacZ.
(TIF)

S12 Fig Loss of *dMyc-* or *scrib*- induced cell death is independent of Pll. (**A**-**E**) Light micrographs showing *Drosophila* adult wings. Compared with control (**A**), RNAi-mediated depletion of *dMyc* driven by *ptc*-Gal4 triggered cell death that resulted in reduced wing area between L3 and L4 (**B**), which is independent of Pll or LacZ (**C**-**E**). Fluorescence micrographs of third instar larval wing discs stained with AO (**G-K**) or anti-Cleaved Caspase-3 (CC-3) antibody (**M-Q**) are shown. Compared with controls (**G** and **M**), loss of *scrib* along the A/P boundary resulted in increased cell death (**H**) and caspase activity (**N**), both of which were not suppressed by up/down-regulation of Pll or expression of LacZ (**I**-**K** and **O**-**Q**). **M’**-**Q’** are high magnification of the boxed areas in **M**-**Q**. (**F**, **L** and **R**) Quantifications of adult wing size L3-L4/total ratio (**F**), cell death with AO staining (**L**) and CC-3 antibody staining (**R**) are shown for figures **A-E, G**-**K** and **M-Q** respectively. One-way ANOVA with Bonferroni multiple comparison test was used to compute *P*-values, significance is indicated with asterisks (*** *P*<0.001). ns stands for not significant. Detailed genotypes: (A, G and M) *ptc-*Gal4/+ (B) *ptc-*Gal4/*UAS-dMyc-IR* (C) *ptc-*Gal4/*UAS-dMyc-IR*; *UAS*-Pll/+ (D) *ptc-*Gal4/*UAS-dMyc-IR*; *pll* ^*7*^/+ (E) *ptc-*Gal4/*UAS-dMyc-IR*; *UAS-*LacZ/+ (H and N) *ptc-*Gal4/*UAS-scrib-IR* (I and O) *ptc-*Gal4/*UAS-scrib-IR*; *UAS*-Pll/+ (J and P) *ptc-*Gal4/*UAS-scrib-IR*; *pll* ^*7*^/+ (K and Q) *ptc-*Gal4/*UAS-scrib-IR*; *UAS-*LacZ/+.
(TIF)
