# Supplementary figures and images for: Pelle Modulates dFoxO-Mediated Cell Death in Drosophila
Source: PLoS Genet. 2015 Oct 16;11(10):e1005589. doi: 10.1371/journal.pgen.1005589 (PMC4608839; doi:10.1371/journal.pgen.1005589)

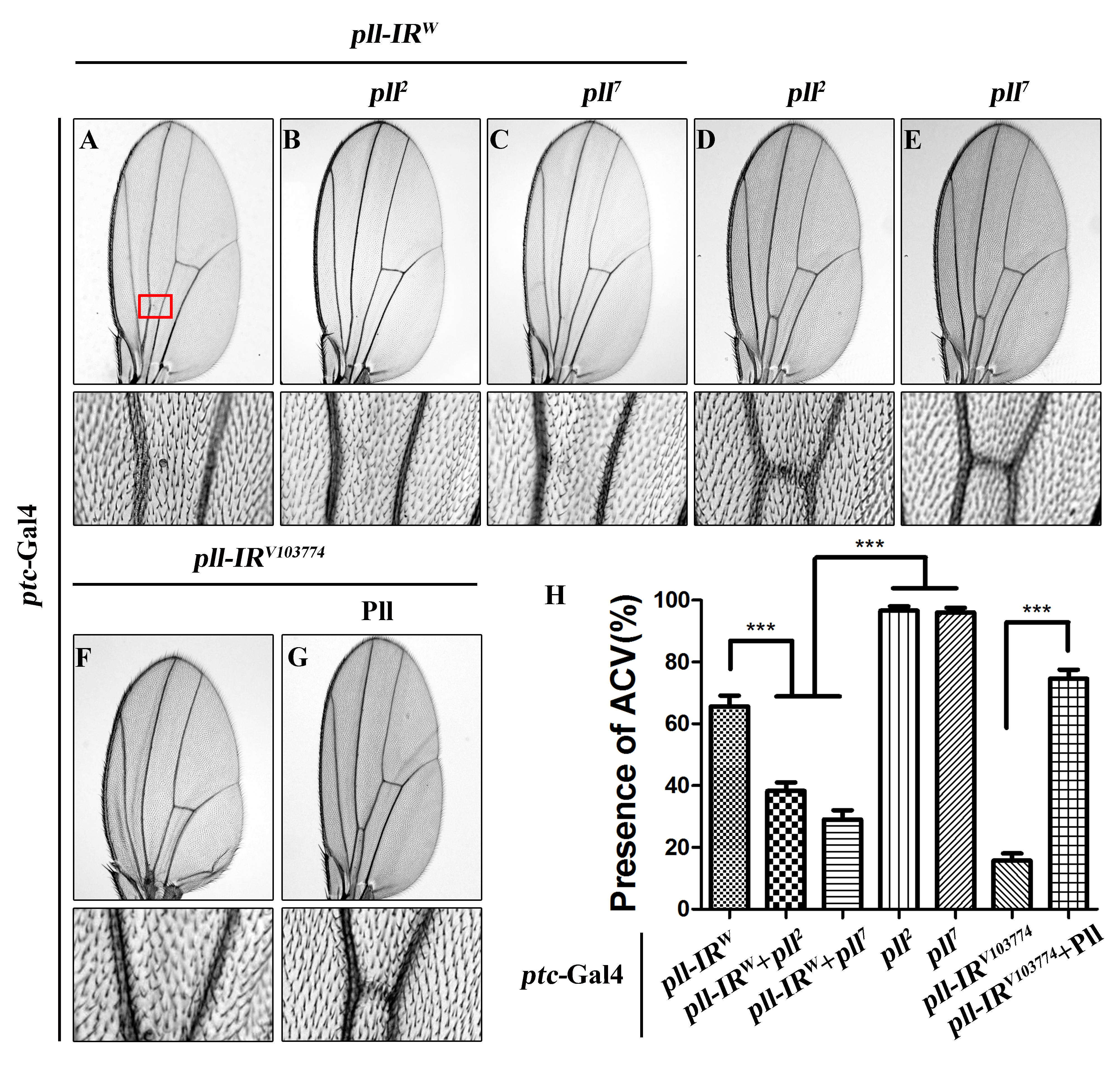

Supplement: S1 Fig — (A-G) Light micrographs showing Drosophila adult wings. Expression of two additional pll RNAi, pll-IR W (A) and pll-IR V103774 (F), driven by ptc-Gal4 generated a loss-of-ACV phenotype, which was rescued by expression of Pll (G). Heterozygous for two pll mutants, pll 2 (D) and pll 7 (E), showed no obvious defects, but strongly enhanced ptc>pll-IR W induced loss-of-ACV phenotype (B and C). The lower panels show high magnification view of the boxed areas in upper panels. (H) Statistical analysis of the ACV phenotype shown in figures A-G. One-way ANOVA with Bonferroni multiple comparison test was used to compute P-values, significance is indicated with asterisks (*** P<0.001). Detailed genotypes: (A) ptc-Gal4/UAS-pll-IR W (B) ptc-Gal4/UAS-pll-IR W; pll 2/+ (C) ptc-Gal4/UAS-pll-IR W; pll 7/+ (D) ptc-Gal4/+; pll 2/+ (E) ptc-Gal4/+; pll 7/+ (F) ptc-Gal4/UAS-pll-IR V103774 (G) ptc-Gal4/UAS-pll-IR V103774; UAS-Pll/+. (TIF) [file pgen.1005589.s001.tif]

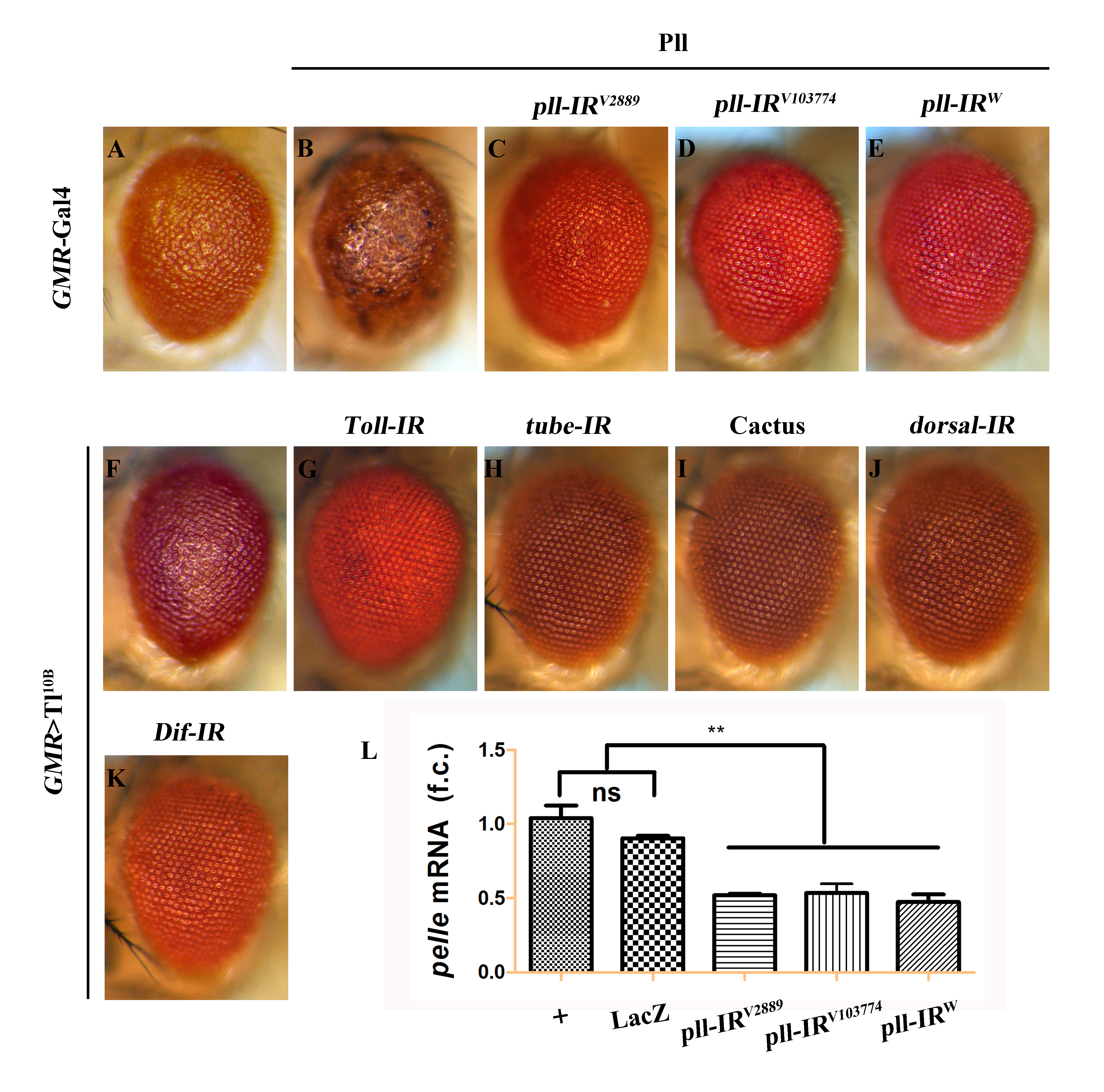

Supplement: S2 Fig — (A-K) Light micrographs of Drosophila adult eyes are shown. Compared with the GMR-Gal4 control (A), GMR>Pll produced a rough eye phenotype (B) that was significantly suppressed by three independent pll RNAi lines (C-E). The rough eye phenotype of GMR>Toll10B (Tl10B) (F) was fully suppressed by RNAi down-regulation of Toll pathway components: Toll, tube, dorsal and Dif, or ectopic expression of Cactus (G-K). (L) The knock-down efficacies of pll RNAi lines. Expression of three pll RNAi, but not LacZ, significantly reduced the level of pll mRNA, as measured by quantitative RT-PCR. Total RNA of Drosophila third instar larvae were extracted and normalized for cDNA synthesis. Error bars represent standard deviation from three independent experiments. Parison test was used to compute P-values, significance is indicated with asterisks. ** P<0.01, ns stands for not significant. Detailed genotypes: (A) GMR-Gal4/+ (B) GMR-Gal4/UAS-Pll (C) UAS-pll-IR V2889/+; GMR-Gal4/UAS-Pll (D) UAS-pll-IRV 103774/+; GMR-Gal4/UAS-Pll (E) UAS-pll-IR W/+; GMR-Gal4/UAS-Pll (F) UAS-Toll10B/+; GMR-Gal4/+ (G) UAS-Toll10B/+; GMR-Gal4/UAS-Toll-IR (H) UAS-Toll10B/+; GMR-Gal4/UAS-tube-IR (I) UAS-Toll10B/UAS-Cactus; GMR-Gal4/+ (J) UAS-Toll10B/+; GMR-Gal4/UAS-dorsal-IR (K) UAS-Toll10B/+; GMR-Gal4/UAS-Dif-IR. (TIF) [file pgen.1005589.s002.tif]

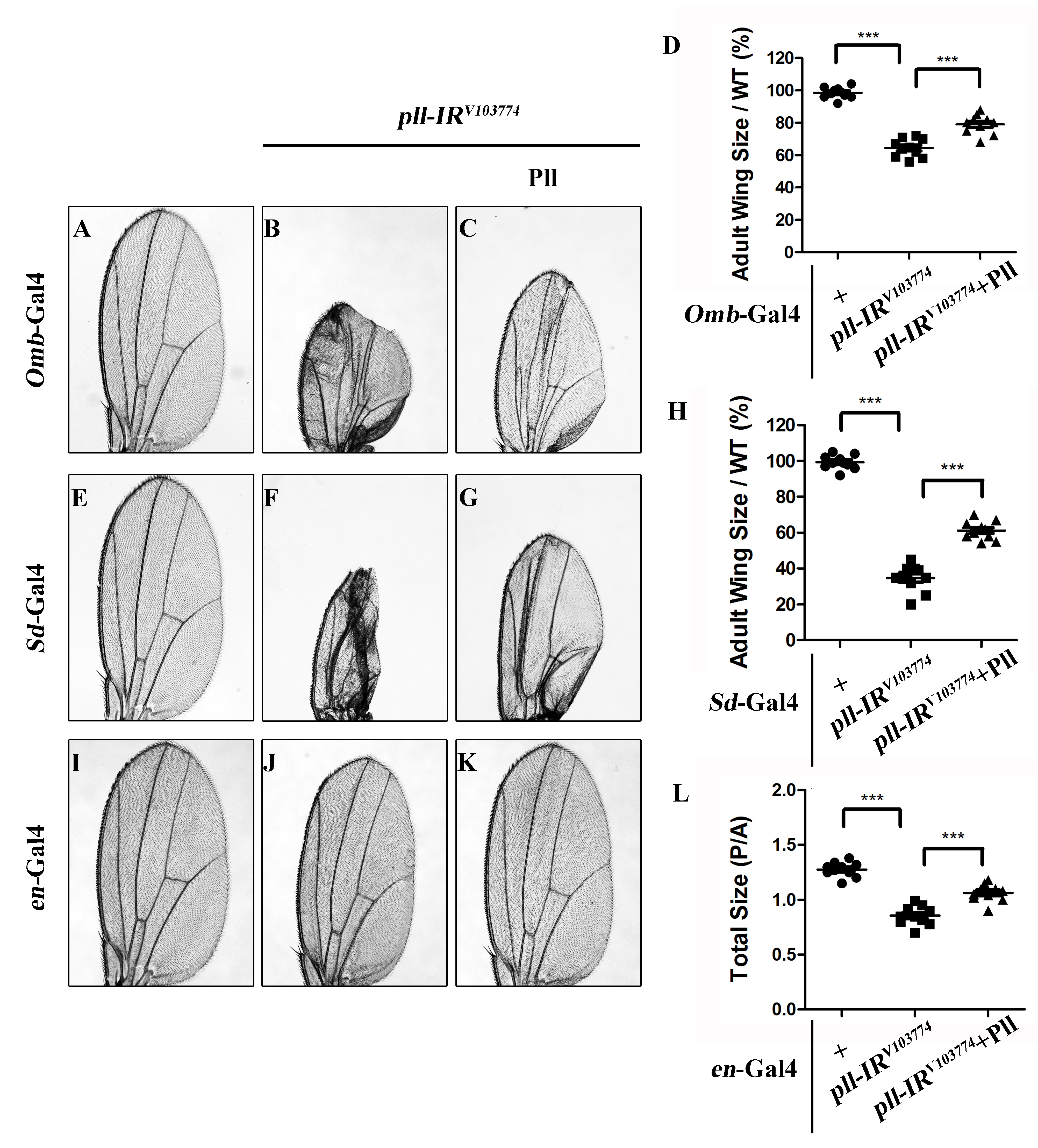

Supplement: S3 Fig — (A-C, E-G and I-K) Light micrographs of Drosophila adult wings are shown. Compared with controls (A, E and I), expression of pll RNAi driven by Omb-Gal4 (B), Sd-Gal4 (F) or en-Gal4 (J) resulted in reduced wing sizes, which were suppressed by expressing Pll (C, G and K). Quantifications of adult wing size/wild type (WT) (D and H) and total size P/A ratio (L) are shown for indicated genotypes. One-way ANOVA with Bonferroni multiple comparison test was used to compute P-values, significance is indicated with asterisks (*** P<0.001). Detailed genotypes: (A) Omb-Gal4/+ (B) Omb-Gal4/+; UAS-pll-IR V103774/+ (C) Omb-Gal4/+; UAS-pll-IR V103774/+; UAS-Pll/+ (E) Sd-Gal4/+ (F) Sd-Gal4/+; UAS-pll-IR V103774/+ (G) Sd-Gal4/+; UAS-pll-IR V103774/+; UAS-Pll/+ (I) en-Gal4/+ (J) en-Gal4/UAS-pll-IR V103774 (K) en-Gal4/UAS-pll-IR V103774; UAS-Pll/+ (TIF) [file pgen.1005589.s003.tif]

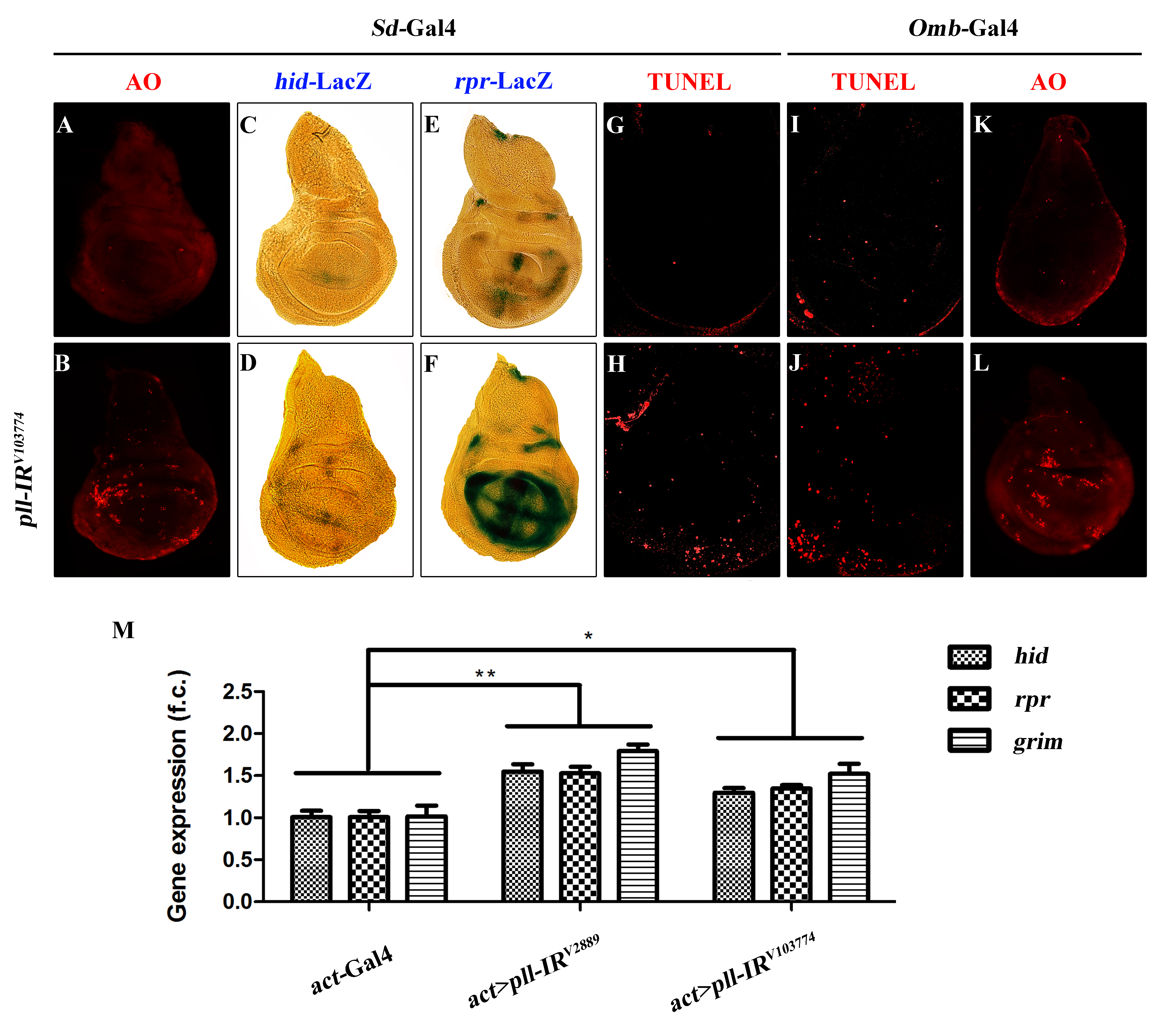

Supplement: S4 Fig — (A, B, K and L) AO staining of third instar larval wing discs. Compared with controls (A and K), knock down pll by Sd-Gal4 (B) or Omb-Gal4 (L) triggered cell death detected by AO staining. (C-F) X-Gal staining of a hid-LacZ and an rpr-LacZ reporters in wing discs. Compared with controls (C and E), knock down pll in the wing pouch induced hid (D) and rpr (F) transcription. (G-J) TUNEL staining of third instar larval wing discs. Compared with Gal4 controls (G and I), knock down pll by Sd-Gal4 (H) or Omb-Gal4 (J) induced cell death in the corresponding areas. In all figures, anterior is to the left and dorsal up. (M) Loss of pll up-regulated the mRNA level of hid, rpr and grim, as measured by quantitative RT-PCR. Total RNA of Drosophila third instar larvae were extracted and normalized for cDNA synthesis. One-way ANOVA with Bonferroni multiple comparison test was used to calculate statistical significance, indicated with asterisks (* P<0.05, ** P<0.01). Error bars represent standard deviation from three independent experiments. Detailed genotypes: (A and G) Sd-Gal4/+ (B and H) Sd-Gal4/+; UAS-pll-IR V103774/+ (C) Sd-Gal4/+; hid-LacZ/+ (D) Sd-Gal4/+; UAS-pll-IR V103774/+; hid-LacZ/+ (E) Sd-Gal4/+; rpr-LacZ/+ (F) Sd-Gal4/+; UAS-pll-IR V103774/+; rpr-LacZ/+ (I and K) Omb-Gal4/+ (J and L) Omb-Gal4/+; UAS-pll-IR V103774/+. (TIF) [file pgen.1005589.s004.tif]

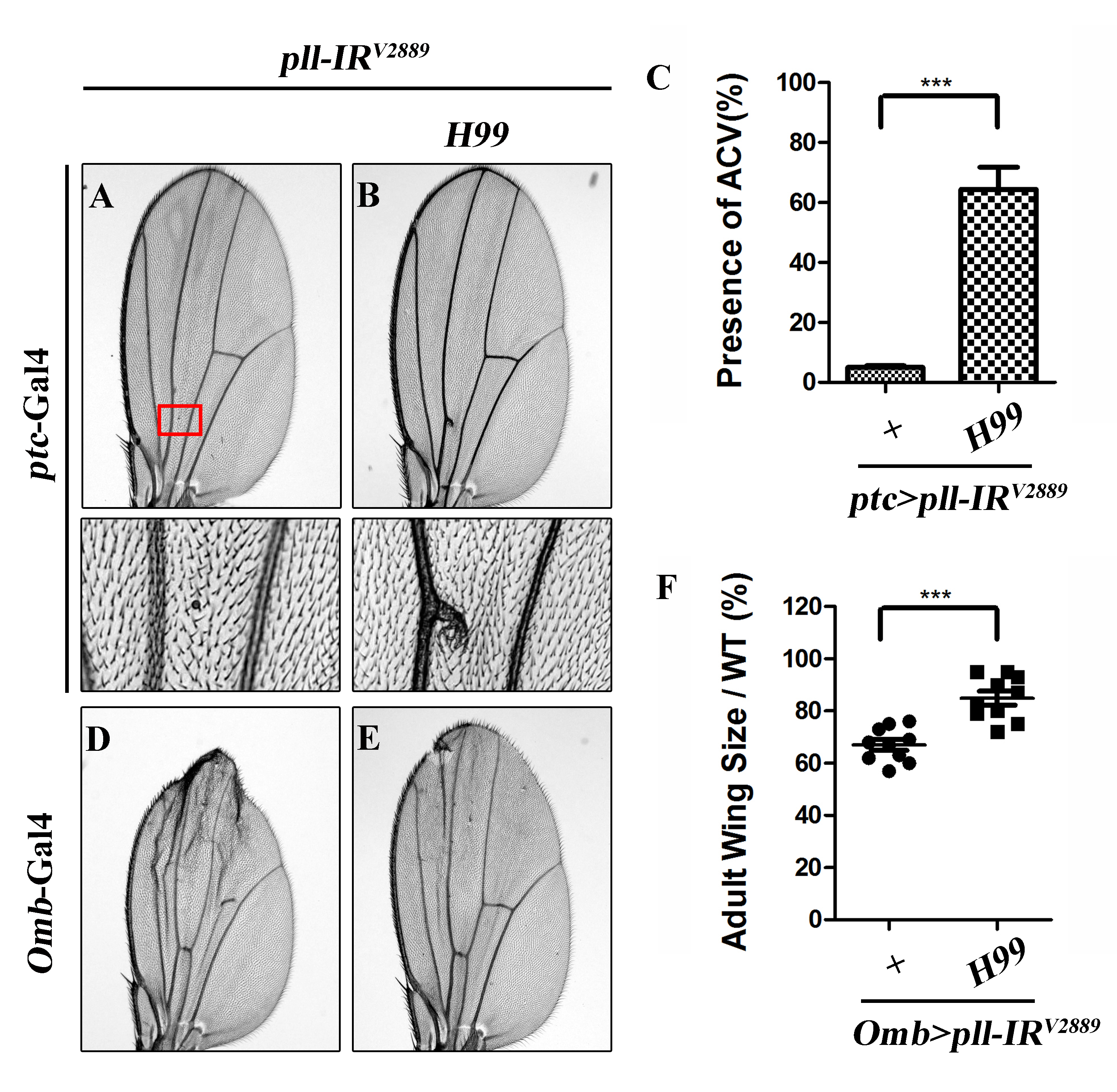

Supplement: S5 Fig — (A, B, D and E) Light micrographs showing Drosophila adult wings. Wing phenotypes of ptc>pll-IR V2889 (A) and Omb>pll-IR V2889 (D) were partially suppressed by Df(3L)H99 that deletes one copy of the apoptotic genes reaper, hid and grim (B and E). The lower panels show high magnification view of the boxed areas in upper panels (A and B). Statistical analysis of the ACV phenotype (C) and quantification of adult wing size/WT (F) are shown for indicated genotypes. Unpaired t test was used to calculate statistical significance, indicated with asterisks (*** P<0.001). Detailed genotypes: (A) ptc-Gal4/UAS-pll-IR V2889 (B) ptc-Gal4/UAS-pll-IR V2889; H99/+ (D) Omb-Gal4/+; UAS-pll-IR V2889/+ (E) Omb-Gal4/+; UAS-pll-IR V2889/+; H99/+. (TIF) [file pgen.1005589.s005.tif]

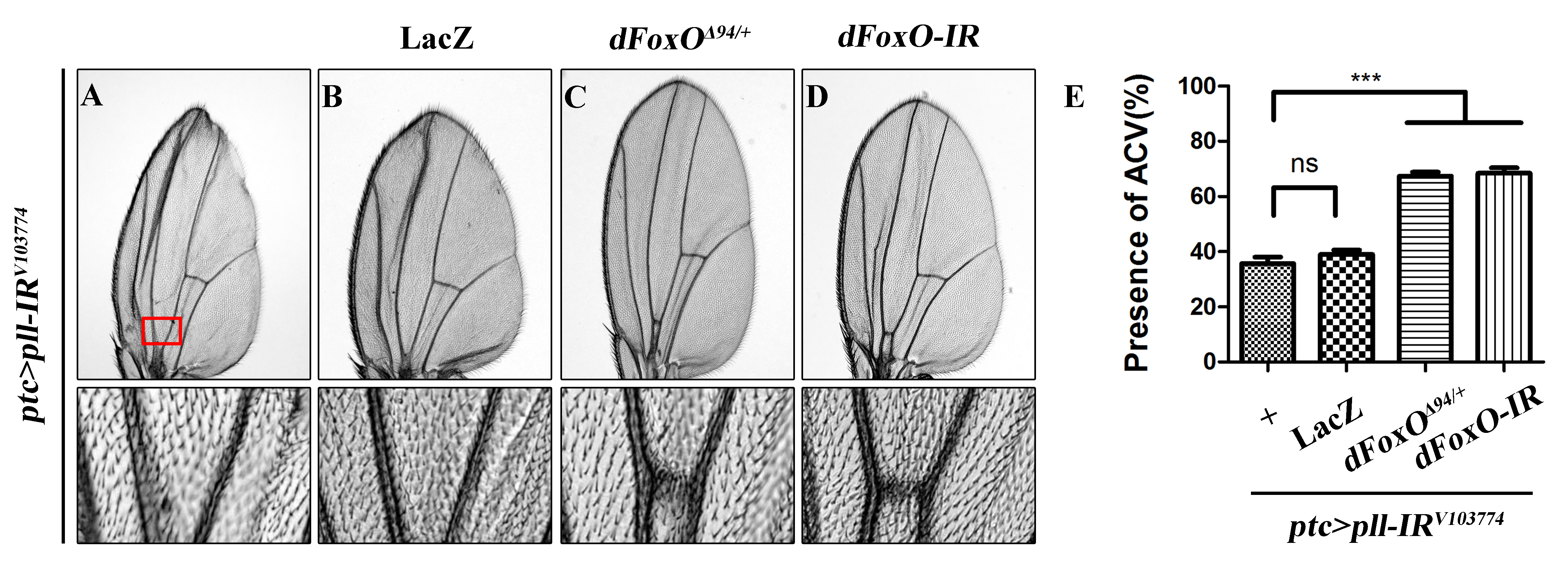

Supplement: S6 Fig — (A-D) Light micrographs showing Drosophila adult wings. The loss-of-ACV phenotype in ptc>pll-IR V103774 flies (A) was suppressed by removing one copy of endogenous dFoxO (C) or expressing a dFoxO RNAi (D), but not by expressing LacZ (B). The lower panels show high magnification view of the boxed areas in upper panels. (E) Quantification of the ACV phenotype as shown in figures A-D. One-way ANOVA with Bonferroni multiple comparison test was used to compute P-values, significance is indicated with asterisks (*** P<0.001). Detailed genotypes: (A) ptc-Gal4/UAS-pll-IR V103774 (B) ptc-Gal4/UAS-pll-IR V103774; UAS-LacZ/+ (C) ptc-Gal4/UAS-pll-IR V103774; dFoxO Δ94/+ (D) ptc-Gal4/UAS-pll-IR V103774; UAS-dFoxO-IR/+. (TIF) [file pgen.1005589.s006.tif]

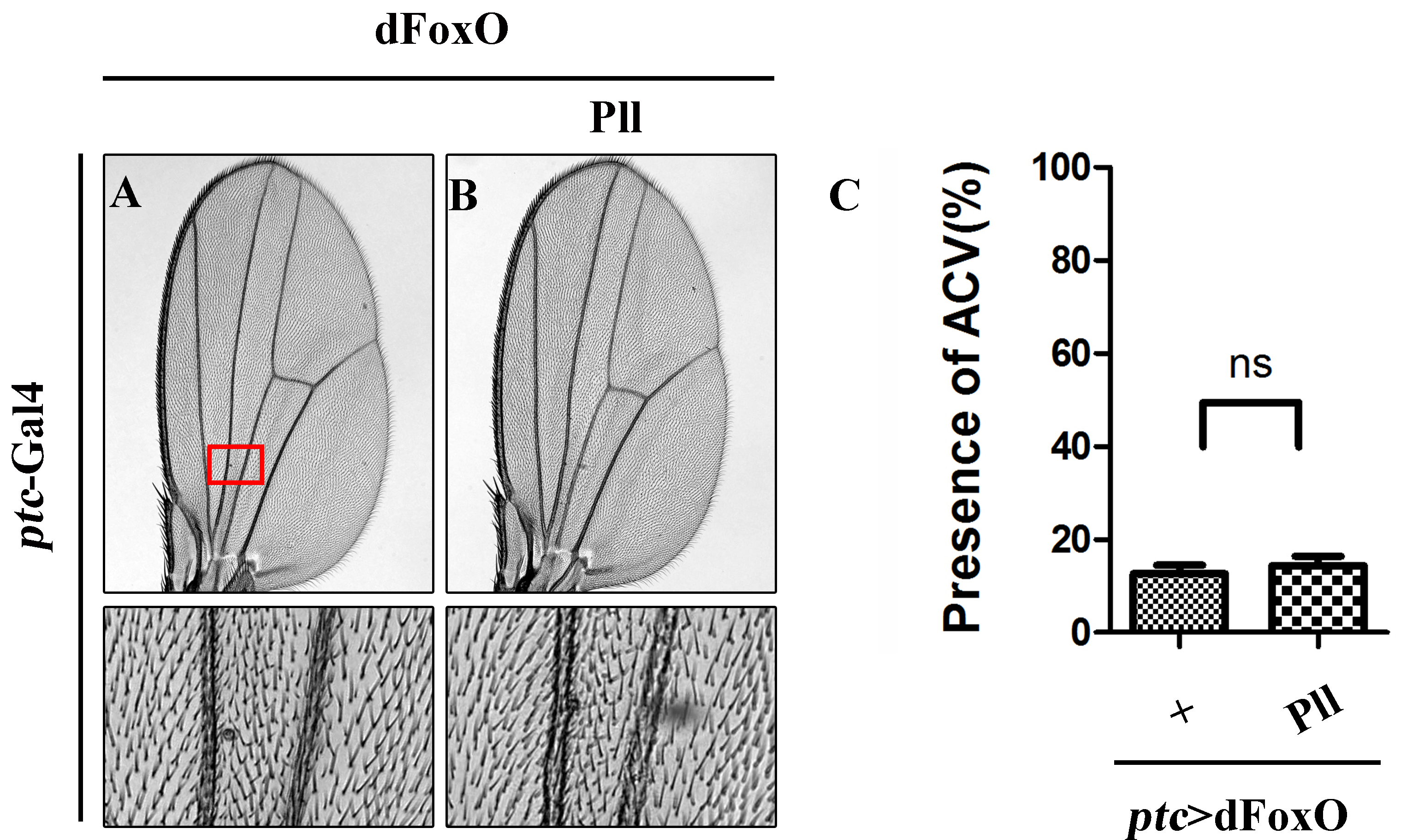

Supplement: S7 Fig — (A and B) Light micrographs of Drosophila adult wings are shown. Expression of dFoxO driven by ptc-Gal4 recapitulated the loss-of-ACV phenotype (A), which was not suppressed by the expression of Pll (B). (C) Statistical analysis of the ACV phenotype shown in figures A and B. Unpaired t test was used to calculate statistical significance. ns stands for not significant. Detailed genotypes: (A) ptc-Gal4/UAS-dFoxO (B) ptc-Gal4/UAS-dFoxO; UAS-Pll/+. (TIF) [file pgen.1005589.s007.tif]

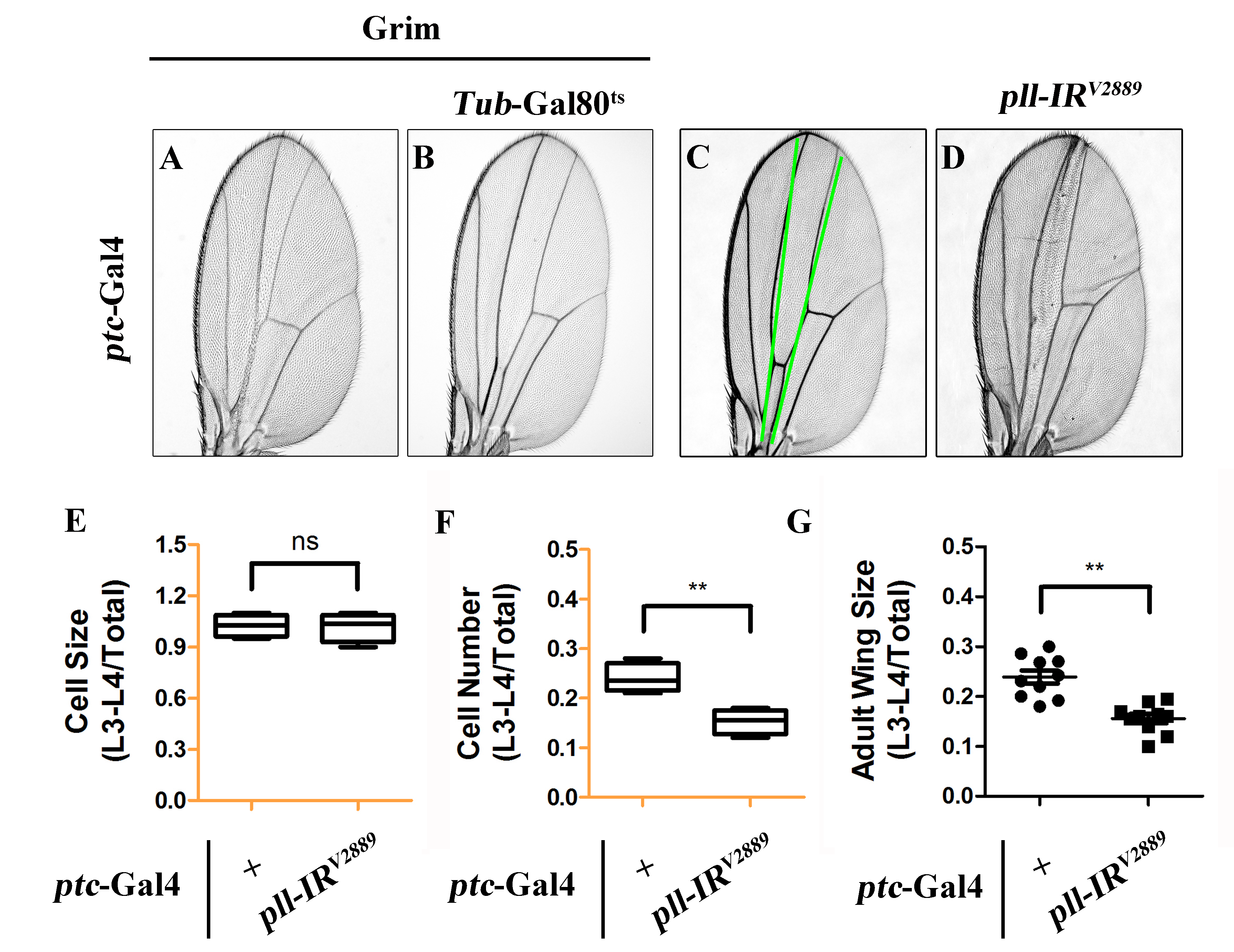

Supplement: S8 Fig — (A-D) Light micrographs showing Drosophila adult wings. Compared with the ptc-Gal4 control (C), expression of Grim triggered strong cell death that resulted in partially fused L3 and L4 (A), while limited Grim expression imposed by Tub-Gal80ts produced a loss-of-ACV phenotype (B). Enhanced expression of a pll RNAi (at 29°C) not only abolished ACV, but also diminished the area between L3 and L4 (D). (E-G) Quantifications of cell size (E), cell number (F) and area size (G) of L3-L4/total ratio in C and D are shown. While cell size was not affected, both cell number and area size decreased significantly when pll was knocked down by ptc-Gal4. Unpaired t test was used to calculate statistical significance, indicated with asterisks (**P<0.01, n = 10 in each group). ns stands for not significant. Detailed genotypes: (A) ptc-Gal4/UAS-Grim (B) ptc-Gal4/UAS-Grim; Tub-Gal80ts/+ (C) ptc-Gal4/+ (D) ptc-Gal4/UAS-pll-IR V2889. (TIF) [file pgen.1005589.s008.tif]

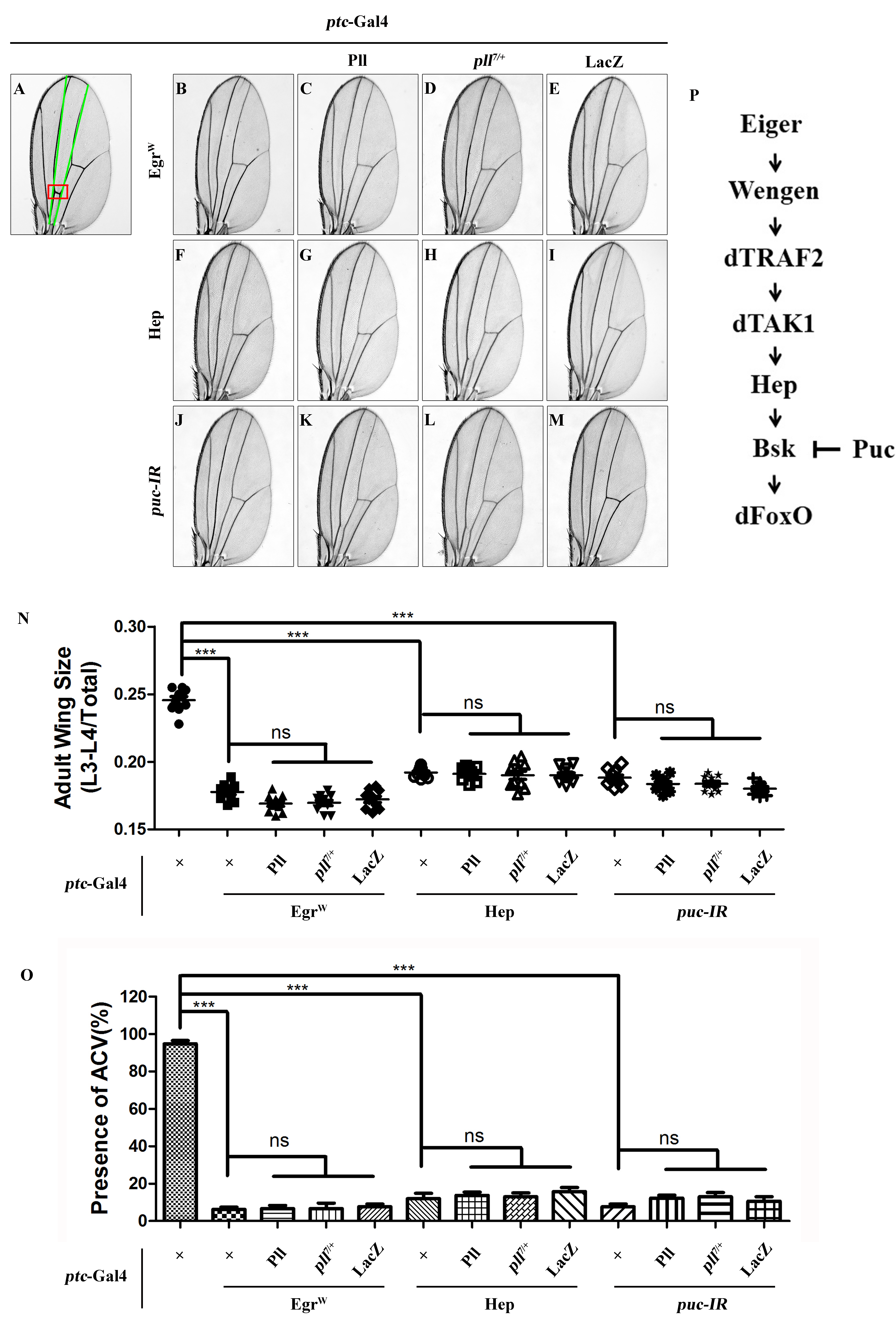

Supplement: S9 Fig — (A-M) Light micrographs of Drosophila adult wings are shown. Compared with ptc-Gal4 control (A), expression of Egrw (B) or Hep (F), or RNAi-mediated down-regulation of puc (J) along the A/P compartment boundary resulted in a loss-of-ACV phenotype and size reduction between L3 and L4. The phenotypes are not affected by gain- or loss-of-pll, or by expression of LacZ (C-E, G-I and K-M). Statistics analysis of area size L3-L4/total ratio (N) and the ACV phenotype (O) are shown for figures A-M. One-way ANOVA with Bonferroni multiple comparison test was used to compute P-values, significance is indicated with asterisks (*** P<0.001). ns stands for not significant. (P) A diagram for the key components of JNK pathway. Detailed genotypes: (A) ptc-Gal4/+ (B) ptc-Gal4/UAS-EgrW (C) ptc-Gal4/UAS-EgrW; UAS-Pll/+ (D) ptc-Gal4/UAS-EgrW; pll 7/+ (E) ptc-Gal4/UAS-EgrW; UAS-LacZ/+ (F) ptc-Gal4/UAS-Hep (G) ptc-Gal4/UAS-Hep; UAS-Pll/+ (H) ptc-Gal4/UAS-Hep; pll 7/+ (I) ptc-Gal4/UAS-Hep; UAS-LacZ/+ (J) ptc-Gal4/UAS-puc-IR (K) ptc-Gal4/UAS-puc-IR; UAS-Pll/+ (L) ptc-Gal4/UAS-puc-IR; pll 7/+ (M) ptc-Gal4/UAS-puc-IR; UAS-LacZ/+. (TIF) [file pgen.1005589.s009.tif]

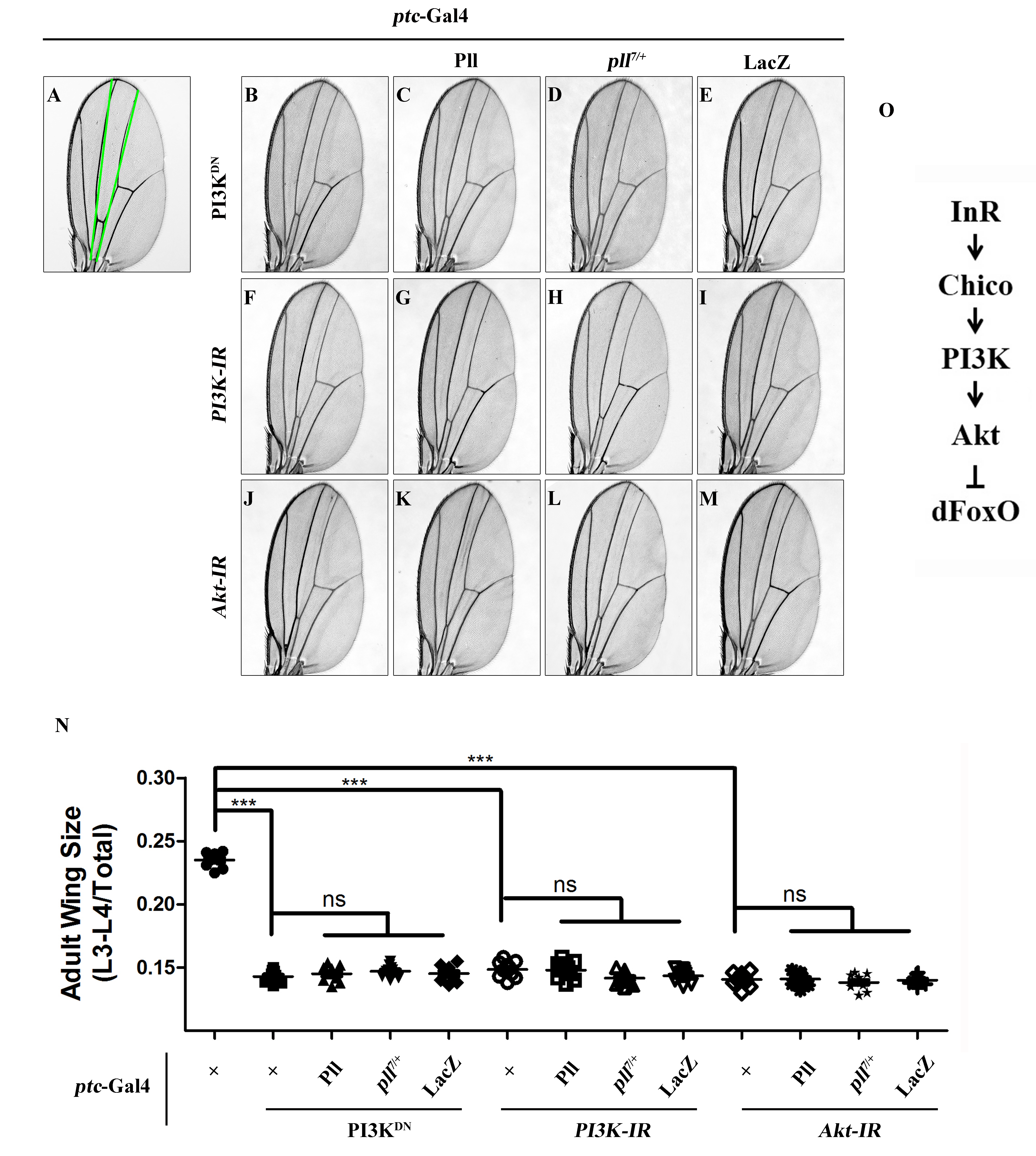

Supplement: S10 Fig — (A-M) Light micrographs showing Drosophila adult wings. Compared with ptc-Gal4 control (A), expression of a dominant negative form of PI3K (B), or RNAi-mediated inactivation of PI3K (F) or Akt (J) led to a size reduction between L3 and L4, which was not affected by changing Pll level or expressing LacZ (C-E, G-I and K-M). (N) Quantification of adult wing size L3-L4/total ratio as shown in figures A-M. One-way ANOVA with Bonferroni multiple comparison test was used to compute P-values, significance is indicated with asterisks (*** P<0.001). ns stands for not significant. (O) A diagram for the key components of Insulin pathway. Detailed genotypes: (A) ptc-Gal4/+ (B) ptc-Gal4/+; UAS-PI3KDN/+ (C) ptc-Gal4/+; UAS-PI3KDN/UAS-Pll (D) ptc-Gal4/+; UAS-PI3KDN/pll 7 (E) ptc-Gal4/+; UAS-PI3KDN/UAS-LacZ (F) ptc-Gal4/+; UAS-PI3K-IR/+ (G) ptc-Gal4/+; UAS-PI3K-IR/UAS-Pll (H) ptc-Gal4/+; UAS-PI3K-IR/pll 7 (I) ptc-Gal4/+; UAS-PI3K-IR/UAS-LacZ (J) ptc-Gal4/UAS-Akt-IR (K) ptc-Gal4/UAS-Akt-IR; UAS-Pll/+ (L) ptc-Gal4/UAS-Akt-IR; pll 7/+ (M) ptc-Gal4/UAS-Akt-IR; UAS-LacZ/+. (TIF) [file pgen.1005589.s010.tif]

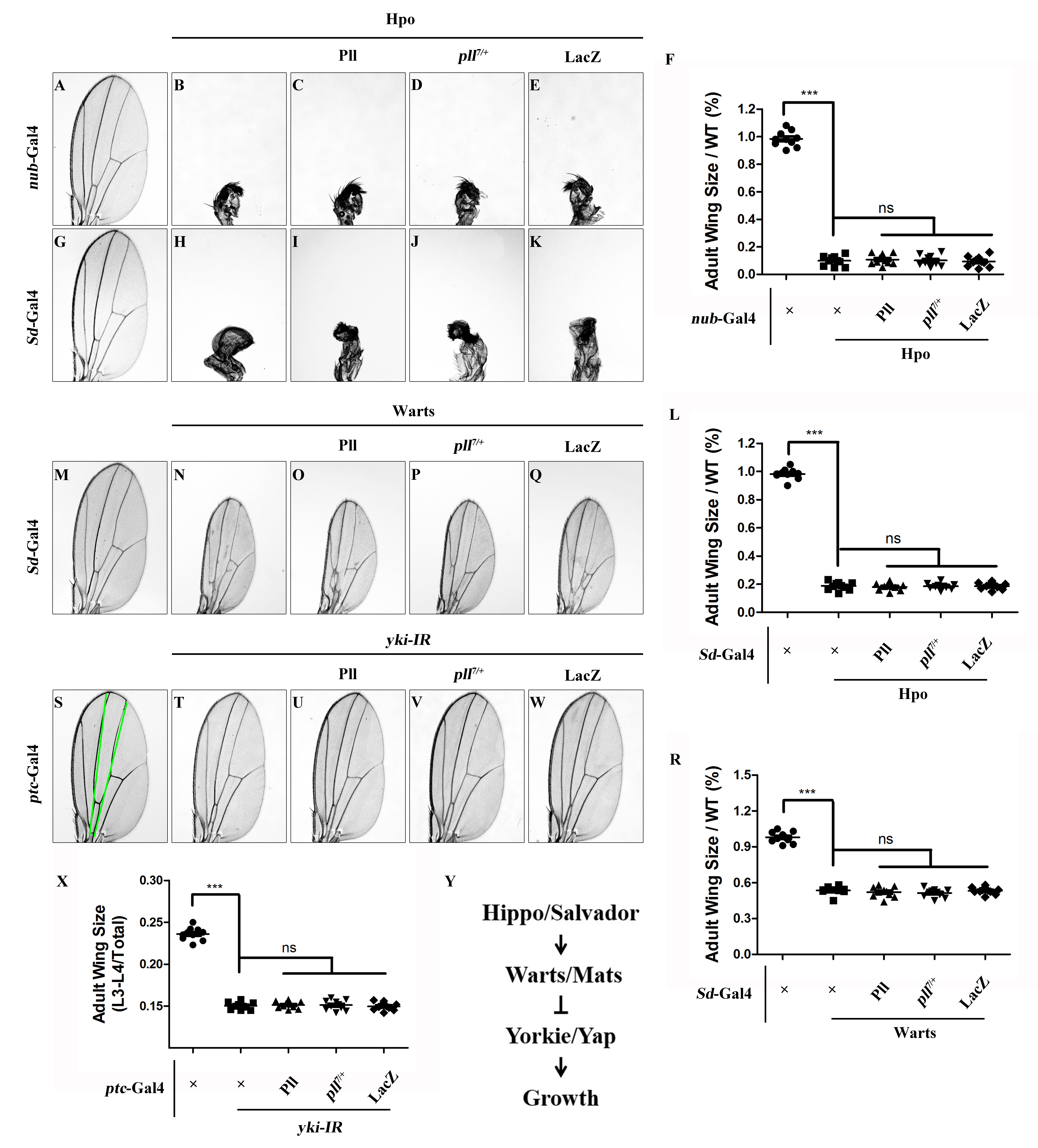

Supplement: S11 Fig — (A-E, G-K, M-Q and S-W) Light micrographs of Drosophila adult wings are shown. Compared with controls (A, G, M and S), up-regulated Hippo signaling by expressing Hippo (Hpo) under the control of nub-Gal4 or Sd-Gal4 (B and H), or expressing Warts by Sd-Gal4 (N), or knocking-down yki by ptc-Gal4 (T) resulted in reduced wing tissue in the corresponding areas, which were not altered by changing Pll level or expressing LacZ (C-E, I-K, O-Q and U-W). (F, L, R and X) Quantifications of total wing size/wild type (WT) ratio or wing size L3-L4/total ratio are shown for figures A-E, G-K, M-Q and S-W respectively (n = 10). One-way ANOVA with Bonferroni multiple comparison test was used to compute P-values, significance is indicated with asterisks (*** P<0.001). ns stands for not significant. (Y) A diagram for the key components of Hippo pathway. Detailed genotypes: (A) nub-Gal4/+ (B) nub-Gal4/+; UAS-Hippo/+ (C) nub-Gal4/+; UAS-Hippo/UAS-Pll (D) nub-Gal4/+; UAS-Hippo/pll 7 (E) nub-Gal4/+; UAS-Hippo/UAS-LacZ (G) Sd-Gal4/+ (H) Sd-Gal4/+; UAS-Hippo/+ (I) Sd-Gal4/+; UAS-Hippo/UAS-Pll (J) Sd-Gal4/+; UAS-Hippo/pll 7 (K) Sd-Gal4/+; UAS-Hippo/UAS-LacZ (M) Sd-Gal4/+ (N) Sd-Gal4/+; UAS-Warts/+ (O) Sd-Gal4/+; UAS-Warts/UAS-Pll (P) Sd-Gal4/+; UAS-Warts/pll 7 (Q) Sd-Gal4/+; UAS-Warts/UAS-LacZ (S) ptc-Gal4/+ (T) ptc-Gal4/+; UAS-yki-IR/+ (U) ptc-Gal4/+; UAS-yki-IR/UAS-Pll (V) ptc-Gal4/+; UAS-yki-IR/pll 7 (W) ptc-Gal4/+; UAS-yki-IR/UAS-LacZ. (TIF) [file pgen.1005589.s011.tif]

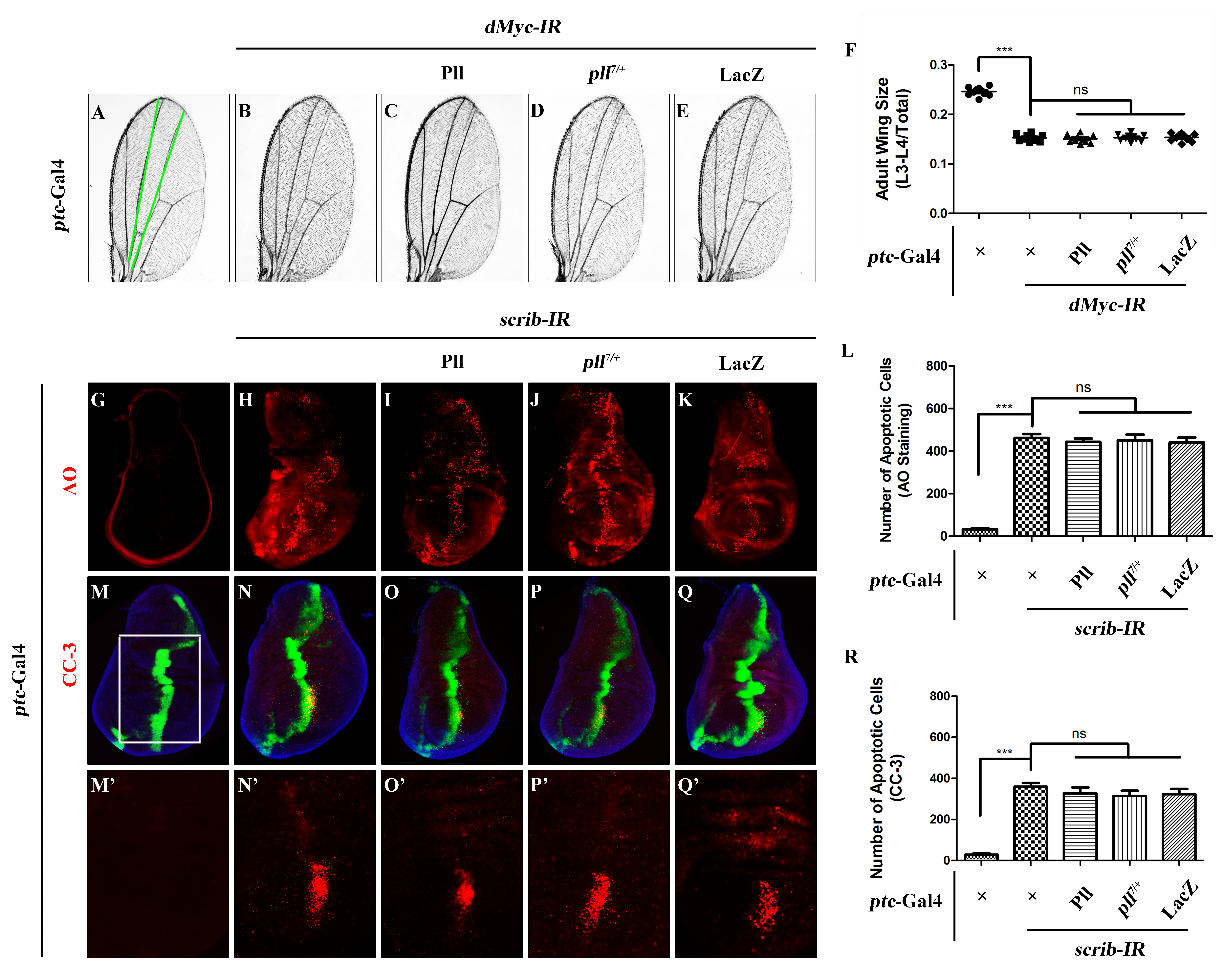

Supplement: S12 Fig — (A-E) Light micrographs showing Drosophila adult wings. Compared with control (A), RNAi-mediated depletion of dMyc driven by ptc-Gal4 triggered cell death that resulted in reduced wing area between L3 and L4 (B), which is independent of Pll or LacZ (C-E). Fluorescence micrographs of third instar larval wing discs stained with AO (G-K) or anti-Cleaved Caspase-3 (CC-3) antibody (M-Q) are shown. Compared with controls (G and M), loss of scrib along the A/P boundary resulted in increased cell death (H) and caspase activity (N), both of which were not suppressed by up/down-regulation of Pll or expression of LacZ (I-K and O-Q). M’-Q’ are high magnification of the boxed areas in M-Q. (F, L and R) Quantifications of adult wing size L3-L4/total ratio (F), cell death with AO staining (L) and CC-3 antibody staining (R) are shown for figures A-E, G-K and M-Q respectively. One-way ANOVA with Bonferroni multiple comparison test was used to compute P-values, significance is indicated with asterisks (*** P<0.001). ns stands for not significant. Detailed genotypes: (A, G and M) ptc-Gal4/+ (B) ptc-Gal4/UAS-dMyc-IR (C) ptc-Gal4/UAS-dMyc-IR; UAS-Pll/+ (D) ptc-Gal4/UAS-dMyc-IR; pll 7/+ (E) ptc-Gal4/UAS-dMyc-IR; UAS-LacZ/+ (H and N) ptc-Gal4/UAS-scrib-IR (I and O) ptc-Gal4/UAS-scrib-IR; UAS-Pll/+ (J and P) ptc-Gal4/UAS-scrib-IR; pll 7/+ (K and Q) ptc-Gal4/UAS-scrib-IR; UAS-LacZ/+. (TIF) [file pgen.1005589.s012.tif]
